# G-quadruplexes sense natural porphyrin metabolites for regulation of gene transcription and chromatin landscapes

**DOI:** 10.1186/s13059-022-02830-8

**Published:** 2022-12-15

**Authors:** Conghui Li, Zhinang Yin, Ruijing Xiao, Beili Huang, Yali Cui, Honghong Wang, Ying Xiang, Lingrui Wang, Lingyu Lei, Jiaqin Ye, Tianyu Li, Youquan Zhong, Fangteng Guo, Yuchen Xia, Pingping Fang, Kaiwei Liang

**Affiliations:** 1 School of Basic Medical Sciences, Wuhan University, Wuhan, 430071 China; 2 State Key Laboratory of Virology and Hubei Province Key Laboratory of Allergy and Immunology, Wuhan University, Wuhan, 430071 China; 3 TaiKang Center for Life and Medical Sciences, TaiKang Medical School, Wuhan University, Wuhan, 430071 China; 4 Hubei Province Key Laboratory of Allergy and Immunology, School of Basic Medical Sciences, Wuhan University, Wuhan, 430071 China

**Keywords:** G-quadruplex, Transcription initiation, Hemin, Porphyrins, G4-CUT&Tag, Chromatin landscapes

## Abstract

**Background:**

G-quadruplexes (G4s) are unique noncanonical nucleic acid secondary structures, which have been proposed to physically interact with transcription factors and chromatin remodelers to regulate cell type-specific transcriptome and shape chromatin landscapes.

**Results:**

Based on the direct interaction between G4 and natural porphyrins, we establish genome-wide approaches to profile where the iron-liganded porphyrin hemin can bind in the chromatin. Hemin promotes genome-wide G4 formation, impairs transcription initiation, and alters chromatin landscapes, including decreased H3K27ac and H3K4me3 modifications at promoters. Interestingly, G4 status is not involved in the canonical hemin-BACH1-NRF2-mediated enhancer activation process, highlighting an unprecedented G4-dependent mechanism for metabolic regulation of transcription. Furthermore, hemin treatment induces specific gene expression profiles in hepatocytes, underscoring the in vivo potential for metabolic control of gene transcription by porphyrins.

**Conclusions:**

These studies demonstrate that G4 functions as a sensor for natural porphyrin metabolites in cells, revealing a G4-dependent mechanism for metabolic regulation of gene transcription and chromatin landscapes, which will deepen our knowledge of G4 biology and the contribution of cellular metabolites to gene regulation.

**Supplementary Information:**

The online version contains supplementary material available at 10.1186/s13059-022-02830-8.

## Background

Eukaryotic messenger RNA expression is highly orchestrated by the concerted action of RNA polymerase II (Pol II), transcription factors, and chromatin landscapes [1–3]. To achieve appropriate developmental decisions or maintain homeostasis, higher organisms have evolved sophisticated mechanisms for coordinating the expression of their genomes to various intra- and extracellular cues [4]. Increasing evidence reveals how small-molecule metabolites are key regulators of gene expression [5, 6]. These metabolites can be used as substrates by chromatin-modifying enzymes to shape chromatin landscapes, or to directly regulate the activities of transcription factors and their cofactors [7–9]. Therefore, Pol II and chromatin plasticity integrate intra- and extracellular metabolic inputs for appropriate gene transcription, constituting a regulatory interface between metabolites to fine-tune gene expression [10, 11].

G-quadruplexes (G4s) are four-stranded nucleic acid structures formed by Hoogsteen hydrogen-bonded guanines and monovalent cations [12, 13]. G4s are typically formed in guanine-rich repeats of nucleic acids and can be conserved across species [14]. In mammalian cells, endogenous G4 structures have been detected and mapped to specific genomic regions, such as telomeres, gene promoters, enhancers, and double-strand break sites [15, 16]. The G4 distribution at these regions is consistent with the demonstrated role of G4 in a variety of cellular processes, including gene transcription, DNA replication and repair, and telomere function [17, 18]. Genome-wide profiling of endogenous G4s in situ or in a chromatin context using next-generation sequencing approaches, reveals cell type- and cell-state-specific G4 landscapes and their direct interplay with gene transcription [19–22]. G4 may upregulate or downregulate individual genes via different mechanisms. G4 could promote or inhibit the recruitment of specific transcription factors and cofactors [23–26], or act as a roadblock directly or indirectly for transcription elongation [13, 27, 28]. In addition, non-template G-quadruplexes have been proposed to facilitate transcription [29]. Despite the numerous studies linking G4 with various aspects of transcriptional control, a better understanding of these activities and the detailed molecular mechanisms of G4 in transcriptional regulation remains a challenge.

G4 status has emerged as a critical switch impacting both physiological and pathological conditions [13]. Depending on the base composition, strand orientation, and loop length, G4 can adopt diverse topologies and undergo flexible conformational changes according to surrounding conditions [30, 31]. G4s can interact with a variety of endogenous proteins including transcription factors, general transcription factors, DNA helicases, and epigenetic and chromatin remodelers to achieve specific biological readouts [17, 32]. Additionally, the planar, hydrophobic structure of G4 is well-suited for small molecules binding to increase or decrease G4 stability; thus, several G4-stabilizing ligands have been developed for potential anticancer treatments [33–35]. For example, G4 ligands have the potential to alter the dynamics of G4, which could inhibit enhancer activation via competition with specific transcription factors for G4 binding [36], or impair transcription initiation at gene promoters through modulation of chromatin states [21].

Mammalian heme and its derivative hemin are ferrous ion- or ferric ion-liganded porphyrins, which are essential cofactors of multiple biological processes containing oxidation, oxygen transportation, and electron transport. High levels of free heme/hemin (referred as “hemin” throughout the text) are toxic to cells due to catalyzed oxidative stress, and intracellular hemin levels are tightly controlled by a fine-tuned balance among biosynthesis, transport, and degradation by heme oxygenases [37, 38]. Hemin levels can impact transcription by inducing the degradation of transcription repressor BTB and CNC homology 1 (BACH1) at antioxidant response elements, and activate the redox-sensitive transcription factor nuclear factor (erythroid-derived 2)-like 2 (NFE2L2, NRF2) through inhibition of Kelch-like ECH-associated protein 1 (KEAP1) [39]. NRF2, in turn, binds antioxidant response elements to activate the transcription of genes involved in iron transport and hemin homeostasis including the heme oxygenase 1 (*HMOX1*) gene [40–42]. Hemin can also enhance GATA-1 activity at some target genes and regulate erythroid cell transcriptome via heme-regulated motifs using BACH1-dependent and BACH1-independent mechanisms [43, 44]. Together, these studies implicate hemin in the regulation of gene transcription through the interaction of transcription factors and DNA motifs.

Hemin is a ligand for G4 in vitro and hemin-G4 structures (G4 DNAzymes) have been actively studied for their catalytic potential [45, 46]. Based on the evidence that the G4 ligand PhenDC3 could displace G4-bound hemin in vitro and induce expression of hemin-responsive genes in cells, G4 was proposed to sequester free hemin in cells as protection from oxidation stress [47]. However, the specificity of hemin distribution in the genome, and its regulatory effects on G4 and transcriptional regulation remain unclear. In this study, we characterized natural porphyrins PpIX and hemin in cells with mass spectrometry and confirmed their direct interactions with G4 by bio-layer interferometry (BLI). We synthesized biotin-PEG4-hemin and established an in situ capture sequencing method using the Cleavage Under Targets and Tagmentation (CUT&Tag) strategy [48, 49] to map the hemin binding sites across the human genome. To map the genome-wide hemin binding sites in the context of chromatin, we combined a G4 self-biotinylation reaction with CUT&Tag. We found that hemin colocalized with G4 peaks and that hemin treatment promoted G4 formation on a genome-wide scale. Hemin treatment inhibited transcription initiation and decreased histone H3K4me3 and H3K27ac modifications at hemin-bound promoters, indicating a role of porphyrin metabolites in the modulation of chromatin landscapes and gene transcription. Furthermore, we found that G4 status was not directly involved in the hemin-BACH1-NRF2-mediated enhancer activation process, suggesting that G4-hemin regulation of transcription is distinct from the canonical transcription factor-mediated pathways [41, 42]. Our findings reveal the essential roles of G4 in the recognition of porphyrins in cells and propose a G4-dependent model for metabolic regulation of gene expression and chromatin landscapes.

## Results

### Characterization of natural porphyrins PpIX and hemin in cells as well as their physical interactions with G4

**Fig. 1:**
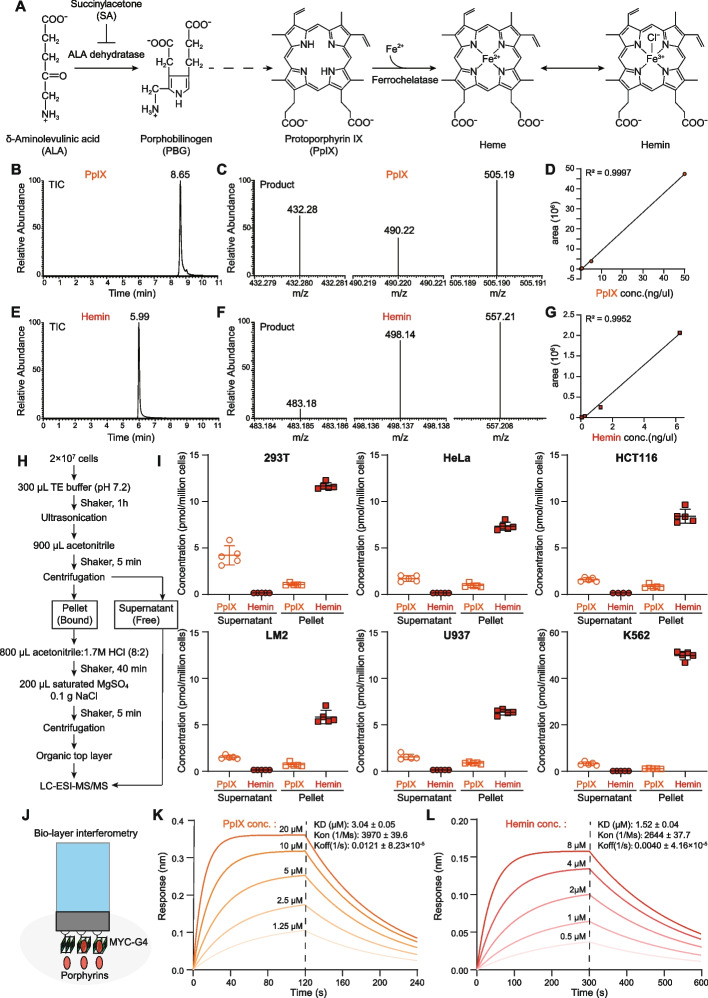
Characterization of natural porphyrins PpIX and hemin in cells and their physical interaction with G4 structures. **A** Scheme of natural porphyrins synthesis from δ-aminolevulinic acid (ALA). **B**,**C** LC-MS/MS analysis of PpIX. Total ion chromatogram (TIC) of PpIX was generated with precursor m/z 564.2 (**B**) and the products m/z were 432.28, 490.22, and 505.19 (**C**). **D** Standard curve for PpIX LC-MS/MS quantification using peak areas. **E**, **F** Mass spectrometry analysis of hemin standards. TIC of hemin was generated with precursor m/z 616 (**E**) and the products m/z were 483.18, 498.14, and 557.21 (**F**). **G** Standard curve for hemin quantification with peak areas using LC-MS/MS analysis. **H** Workflow for extraction and quantification of PpIX and hemin. Collected cells were disrupted and extracted with acetonitrile for free porphyrins. The pellets containing chromatins and insoluble proteins were further hydrolyzed with acid and extracted with acetonitrile for bound porphyrins. **I** Quantification of PpIX and hemin in HEK293T, HeLa, HCT116, LM2, U937, and K562 cells. **J** Characterization of G4-porphyrins interaction with Bio-layer interferometry assays. Biotinylated MYC-G4 was immobilized on streptavidin biosensors and incubated with a range of PpIX or hemin to measure the response in a Gator instrument. **K**, **L** Bio-layer interferometry analysis of MYC-G4 with PpIX (**K**) and hemin (**L**). The dissociation constant KD values for MYC-G4-PpIX and MYC-G4-hemin were 3.04±0.05 μM and 1.52±0.04 μM, respectively

Mammalian porphyrins are generated from δ-aminolevulinic acid (ALA) through several consecutive enzymatic reactions to protoporphyrin IX (PpIX), which is further complexed with an iron cation to produce heme or its derivative hemin (Fig. 1A) [37, 50]. To investigate the relationship between G4 and natural porphyrins in cells, we first characterized the intracellular PpIX and hemin content using liquid chromatography-mass spectrometry (LC-MS/MS) (Fig. 1B–G) [51]. Free porphyrins and bound porphyrins were sequentially extracted from cells for quantitative determination (Fig. 1H) [52]. Six cell lines of different origins, including embryonic kidneys cells (HEK293T), cervical cancer cells (HeLa), colon cancer cells (HCT116), breast cancer cells (MBD-231-LM2), myeloid leukemia cells (U937), and chronic myelogenous leukemia cells (K562), were used for quantification of intracellular PpIX and hemin. PpIX and hemin are present across cell lines but their levels are variable (PpIX: ~0.5–1.2 μM; hemin: ~1.5–12 μM) (Fig. 1I). The majority of PpIX was detected in the supernatant fractions, suggesting that PpIX exists predominately in the free form (Fig. 1I). Consistent with a recent study that G4 could sequester free hemin to protect from oxidation stress [47], we found that the majority of hemin was extracted from the pellets containing the chromatin fractions and insoluble proteins (Fig. 1I).

To characterize the interaction between G4 and porphyrins, real-time binding of PpIX and hemin to biotin-labeled parallel MYC-G4 was monitored by bio-layer interferometry (BLI) (Fig. 1J), revealing a 3.04±0.05 μM dissociation constant (KD) for the complexes of PpIX:MYC-G4 (Fig. 1K), and 1.52±0.04 μM for hemin: MYC-G4 (Fig. 1L), respectively. Hemin showed no significant binding with single-stranded DNA (Additional file 1: Fig. S1A), double-stranded DNA (Fig. S1B), antiparallel SPB1-G4 (Fig. S1C), mixed parallel/antiparallel hTELO-G4 (Fig. S1D), or intermolecular hTELO-G4 (Fig. S1E) [15]. These results evoke the possibility that the specific hemin-G4 interactions can regulate gene transcription and chromatin landscapes.

### Genome-wide profiling of hemin binding sites in the chromatin

**Fig. 2:**
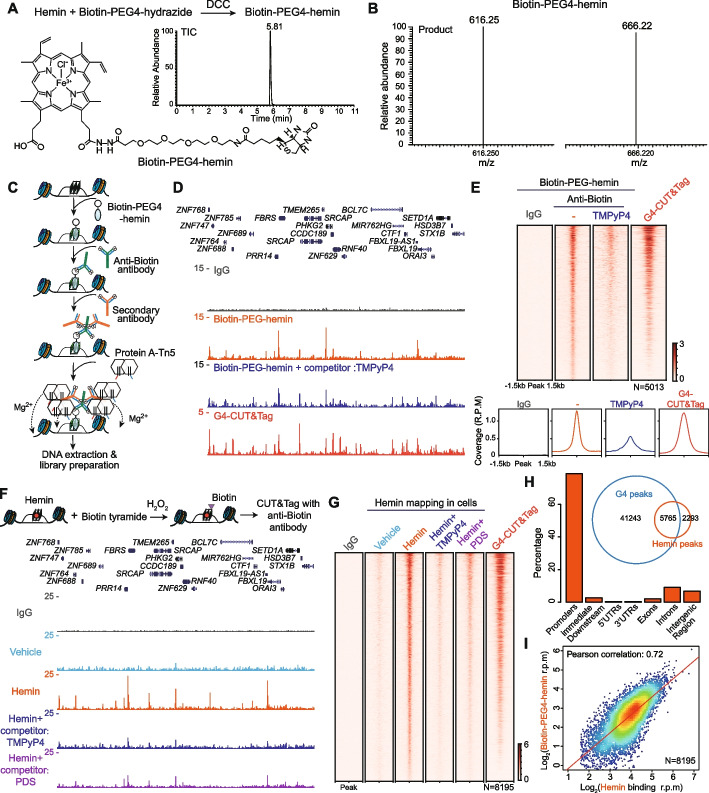
Genome-wide profiling of hemin binding sites using biotin-PEG4-hemin and G4 self-biotinylation reaction. **A**, **B** Synthesis and characterization of biotin-PEG4-hemin. With the coupling reagent dicyclohexyl carbodiimide (DCC), biotin-PEG4-hydrazide reacts with hemin to produce biotin-PEG4-hemin (**A**). TIC of biotin-PEG4-hemin was created with the precursor of m/z 1103.45 (**A**) and the products m/z were 616.25 and 666.22 (**B**). **C** Schematic diagram of profiling of hemin binding sites in the genome using CUT&Tag and biotin-PEG4-hemin. Isolated nuclei were conjugated to concanavalin A-coated magnetic beads, biotin-PEG4-hemin was used to bind with chromatin in situ. Anti-biotin antibody and secondary antibody were added sequentially to tether protein A-Tn5 transposase. After activation with magnesium, Tn5 cut the chromatin close to biotin-PEG4-hemin binding sites and simultaneously integrated to adapters. Then Tn5-tagmented genomic DNA was extracted and amplified for library preparation and second-generation sequencing. For negative controls, IgG was used to replace anti-biotin antibody. **D** UCSC genome browser snapshots of in situ biotin-PEG4-hemin binding signals and G4-CUT&Tag signals in HEK293T cells. Twenty micromolars TMPyP4 was used as a competitor for biotin-PEG4-hemin. **E** Heatmap and metaplot analysis of biotin-PEG4-hemin binding signals and G4-CUT&Tag signals. A total of 5013 biotin-PEG4-hemin binding peaks were identified to be sensitive to TMPyP4 treatment and colocalized with G4. **F** Profiling of hemin binding sites in cells using G4 self-biotinylation reaction and CUT&Tag. UCSC genome browser snapshots of G4 self-biotinylation signals in HEK293T cells in the presence or absence of 80 μM TMPyP4 or PDS competitors. **G** Heatmaps of G4 self-biotinylation in the HEK293T after treatment of vehicle or hemin. With the TMPyP4 competitor, we characterized 8195 G4 self-biotinylation peaks that were sensitive to TMPyP4 treatment. **H** Genome-wide annotation of the 8195 G4 self-biotinylation peaks in HEK293T cells. The Venn diagram shows the overlap of G4-CUT&Tag peaks with the 8195 hemin binding peaks. **I** Pearson’s correlation of in vivo G4 self-biotinylation signals and in situ biotin-PEG4-hemin binding signals at the 8195 G4 self-biotinylation peaks

To identify potential hemin binding sites in the human genome, we synthesized biotin-PEG4-hemin through the condensation reaction of hemin and biotin-PEG4-hydrazide (Fig. 2A). Following its validation by LC-MS/MS (Fig. 2A,B) and dot blot assay (Additional file 1: Fig. S2A; Additional file 2), biotin-PEG4-hemin was integrated into the CUT&Tag approach [48, 49] for genome-wide profiling of hemin (Fig. 2C). In this method, biotin-PEG4-hemin was used to capture DNA fragments in situ, followed by tagmentation with assembled Tn5 transposome for library preparation and sequencing (Additional file 1: Fig. S2B). University of California Santa Cruz (UCSC) genome browser snapshots demonstrate the enrichment of biotin-PEG4-hemin binding signals at specific genomic regions, which overlapped with G4-CUT&Tag signals [21] to some extent (Fig. 2D). TMPyP4, a well-established G4 ligand [53], was used to compete with biotin-PEG4-hemin and it effectively reduced the biotin-PEG4-hemin binding signals (Fig. 2D). Using the TMPyP4 competitor as a control, we identified 5013 biotin-PEG4-hemin binding peaks in HEK293T cells (Fig. 2E), which overlapped with G4-CUT&Tag signals and were sensitive to TMPyP4 treatment. We also identified in situ biotin-PEG4-hemin binding sites in HeLa cells (Additional file 1: Fig. S2C-D). G4Hunter predicted 93,480 G4 motifs within the 21,395 hemin binding peaks, which is 7.5-fold greater than those predicted from equivalent random sequences (Additional file 1: Fig. S2E). Approximately 37.9% (18,218 in 48,069) G4-CUT&Tag peaks overlapped with hemin binding peaks in HeLa cells (Additional file 1: Fig. S2F-G). Moreover, motif analysis of hemin binding peaks in HeLa cells revealed the presence of G-rich motifs (Additional file 1: Fig. S2H).

G4-hemin complexes have intrinsic peroxidase activity and treating G4-hemin complexes with hydrogen peroxide and biotin tyramide can lead to efficient covalent biotinylation of G4 in vitro and in vivo [54] in a highly specific and spatially restricted manner [55]. Therefore, we developed an assay combining G4 self-biotinylation and the CUT&Tag approach to map endogenous hemin binding sites in HEK293T cells (Fig. 2F). UCSC genome browser tracks demonstrate the enrichment of G4 self-biotinylation signals at specific genomic regions (Fig. 2F), which overlap with biotin-PEG-hemin binding sites and G4-CUT&Tag signals (Fig. 2D). These G4 self-biotinylation signals are sensitive to TMPyP4 or Pyridostatin (PDS) treatments (Fig. 2F, G). Using the TMPyP4 competitor as a control, we identified 8195 hemin binding sites in HEK293T cells (Fig. 2G). Annotation of these peaks showed that nearly 80% of them were distributed at promoter regions (*N*=5,858), with most others occupying intronic and intergenic regions (*N*=612) (Fig. 2H). About 72% (5,902 in 8,195) of hemin binding peaks overlapped with G4-CUT&Tag peaks in HEK293T cells (Fig. 2H). Comparing the biotin-PEG-hemin binding signals and G4 self-biotinylation signals revealed a positive correlation at the 8195 hemin binding peaks (*r*=0.72) (Fig. 2I). Together, these results demonstrate that hemin can bind 12.26% (5765 in 47,008) G4 peaks in HEK293T cells (Fig. 2H), revealing a potential role of hemin in G4 regulation.

### Hemin treatment rapidly promotes genome-wide G4 formation

**Fig. 3:**
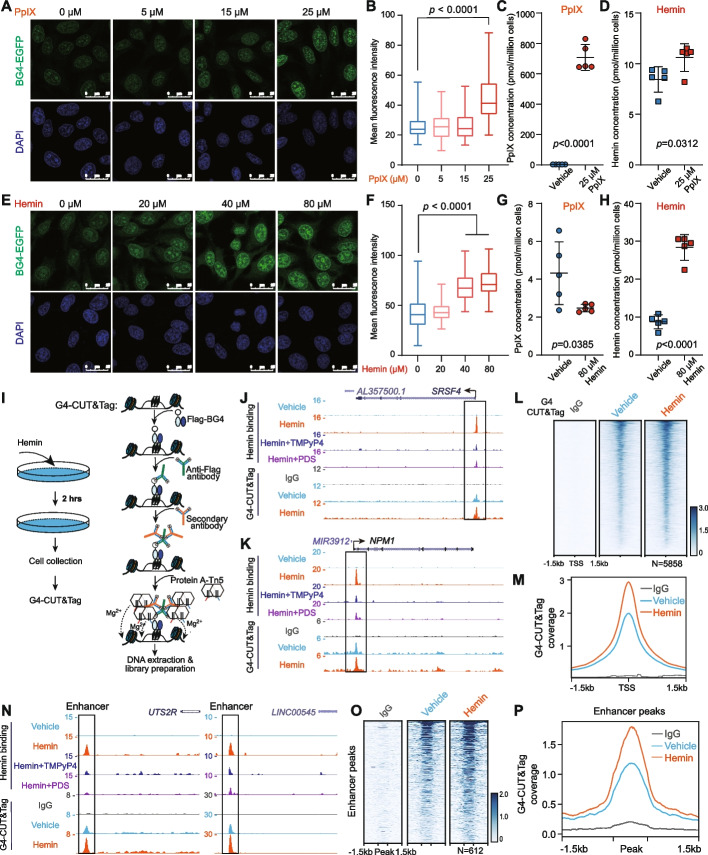
Hemin treatment promotes genome-wide G4 formation. **A**,** B** Immunostaining of G4 using BG4-EGFP (green) in HeLa cells treated with different concentrations of PpIX for 2 h. The nuclei were stained with DAPI (blue) (**A**). For each sample, 139 nuclei in 6 fields of vision were randomly picked, and the mean fluorescence intensities of G4s in each nuclear area were calculated by dividing the total intensities of BG4-EGFP with the nuclear area marked by DAPI (**B**). (10.6084/m9.figshare.21608100.v1) [57]. **C**, **D** Quantification of total PpIX (**C**) and hemin (**D**) in HEK293T cells treated with or without 25 μM PpIX for 2 h. PpIX treatment elevated intracellular PpIX and its downstream product hemin. **E**,** F** Immunostaining of G4 in HeLa cells treated with hemin for 2 h (**E**). For each sample, images of 6 fields of vision were acquired randomly and 135 nuclei were picked for analysis (**F**). **G**, **H** Quantification of total PpIX (**G**) and hemin (**H**) in HEK293T cells treated with or without 80 μM hemin for 2 h. **I** Workflow of G4-CUT&Tag [21] with hemin-treated cells. Following hemin treatment for 2 h, HEK293T cells were collected and used for isolation of nuclei. These nuclei were incubated with Flag-BG4 to recognize G4 in situ. G4 DNA structures were further tagmented by protein A-Tn5 transposase and were amplified for sequencing. **J–M** Analysis of hemin binding promoters and associated G4-CUT&Tag signals in HEK293T cells with or without hemin treatment reveals that hemin increased G4 formation at hemin binding promoters. Track examples at the *SRSF4* (**J**) and *NPM1* (**K**) loci, heatmap (**L**), and metaplot (**M**) are shown. **N–P** Hemin treatment induced an increase of G4 signals at hemin-bound enhancers, as shown in the snapshots (**N**), heatmap (**O**), and metaplot (**P**)

We next treated HeLa cells with PpIX and hemin for a short period of 2 h and determined their effects on G4 levels. Immunostaining of G4 with specific G4 antibody (BG4-EGFP) (Additional file 1: Fig. S3A-B; Additional file 2) [21, 56] showed that PpIX treatment rapidly resulted in increased nuclear G4 levels (Fig. 3A-B; Additional file 1: Fig. S3C), and elevated intracellular PpIX, with a lesser increase of its downstream product hemin as assessed by LC-MS/MS analysis (Fig. 3C,D). We also obtained dose-dependent increases in G4 levels after 2-h hemin treatments (Fig. 3E,F; Additional file 1: Fig. S3C). LC-MS/MS analysis confirmed an increase of intracellular hemin levels from ~2.1 to 6.8 μM after 80 μM hemin treatments, whereas PpIX levels were slightly decreased, which is likely caused by negative feedback (Fig. 3G,H). Treatment with the precursor of porphyrins, ALA, led to increased G4 formation, which could in turn be blocked by succinylacetone (SA), an inhibitor of the rate-limiting ALA dehydratase (ALAD) (Fig. 1A and S3D-E).

To identify changes in G4 genome-wide in response to hemin treatment, we performed G4-CUT&Tag assays [58] in HEK293T cells (Fig. 3I). Two-hour hemin treatment promoted G4 formation at hemin-bound promoters as illustrated in the genome browser views of the *SRSF4* and *NPM1* loci (Fig. 3J,K). Genome-wide analyses show that hemin treatment increased G4-CUT&Tag signal at hemin binding promoters (*N*=5858) (Fig. 3L,M) and at hemin binding enhancer regions (*N*=612) (Fig. 3N,P). Furthermore, since G4 can be structurally compatible with and can colocalize with R-loops, noncanonical triple-stranded nucleic acid structures composed of a DNA: RNA hybrid and a displaced single-stranded DNA [59, 60], we performed R-loop CUT&Tag with the sensor GST-His_6_-2×HBD [61] in HEK293T cells after 2-h hemin treatment to determine if R-loops are affected by hemin (Additional file 1: Fig. S3F). Genome-wide analysis of R-loop signals (Additional file 1: Fig. S3G-J) revealed that hemin treatment decreased R-loop CUT&Tag signals at hemin binding promoters (*N*=5858) and enhancers (*N*=612).

### Impairment of gene transcription initiation by hemin

**Fig. 4:**
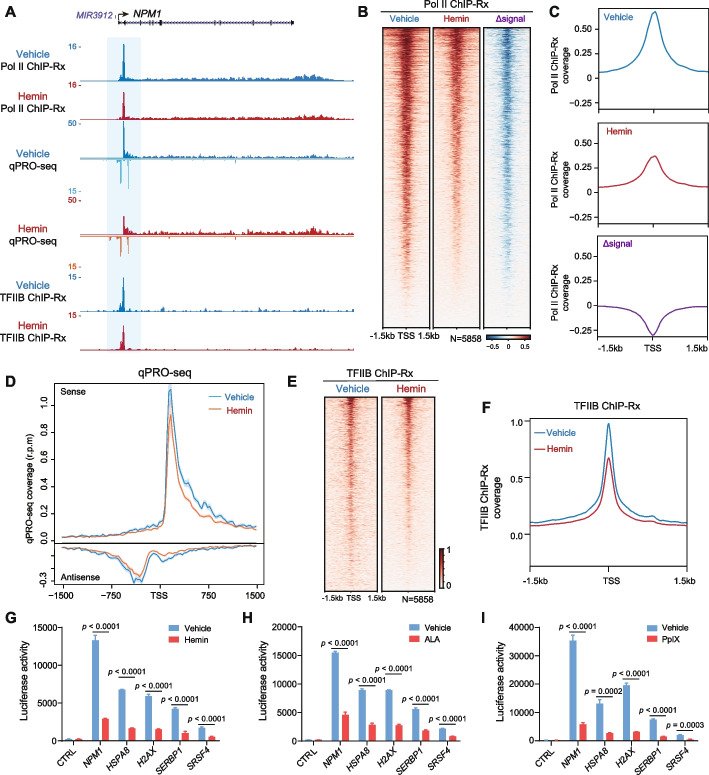
Hemin inhibits transcription initiation at hemin-bound promoters. **A** UCSC genome browser tracks of Pol II ChIP-Rx, qPRO-seq, and TFIIB ChIP-Rx signals at the *NPM1* locus in HEK293T cells. **B,C** Heatmap (**B**) and metaplot (**C**) analyses of Pol II ChIP-Rx signals at hemin-bound promoters demonstrate that hemin impeded Pol II occupancy at hemin-bound promoters. **D** Metaplot of strand-specific qPRO-seq signals at hemin-bound promoters. **E**, **F** Heatmap (**E**) and metaplot (**F**) analyses of TFIIB ChIP-Rx signals at hemin-bound promoters illustrate that hemin treatment impaired general transcription factor TFIIB occupancy at promoters. **G–I** Luciferase reporter assay of G4-containing promoters showed that hemin (**G**), ALA (**H**), and PpIX (**I**) treatments reduced luciferase reporter gene expression

To investigate whether hemin could regulate gene transcription through G4, we performed Pol II ChIP with reference exogenous genome (ChIP-Rx) and quick Precision Run-On sequencing (qPRO-seq) [62] in HEK293T cells after 2-h hemin treatment (Fig. 4A). Genome browser snapshots at the *NPM1* promoter illustrate that hemin decreases Pol II occupancy and qPRO-seq signal intensities, the latter of which represents elongating Pol II (Fig. 4A). Genome-wide analysis of Pol II ChIP-Rx confirmed that hemin treatment led to reduced Pol II occupancy at the 5858 hemin-bound promoters (Fig. 4B,C). Metaplots of strand-specific qPRO-seq signals showed that hemin treatment led to reduced levels of elongating Pol II at these promoters as well (Fig. 4D). To determine whether hemin impairs transcription initiation through a similar mechanism as the G4 ligand TMPyP4, i.e., by impeding general transcription factor loading at promoters [21], we performed ChIP-Rx with an antibody against the general transcription factor TFIIB (Fig. 4A). Genome-wide analysis shows that hemin treatment impaired TFIIB recruitment to hemin-bound promoters (*N*=5858) (Fig. 4E,F), suggesting that hemin could inhibit transcription initiation by impeding the loading of TFIIB and Pol II to promoters.

We further tested the effects of hemin treatment using plasmid templates and reporter gene assays. Recombinant firefly luciferase reporter plasmids were inserted with *NPM1*, *HSPA8*, *H2AX*, *SERBP1*, or *SRSF4* promoters, which can interact with hemin and have previously been shown to form G4s in HEK293T cells [21]. Luciferase activities were measured after treatment with hemin, PpIX, or their precursor ALA, respectively. Consistent with our previous findings with TMPyP4 [21], hemin, PpIX, or ALA treatments attenuated promoter activities of *NPM1*, *HSPA8*, *H2AX*, *SERBP1*, and *SRSF4* (Fig. 4G–I), supporting the notion that hemin impairs transcription initiation at these hemin binding and G4 forming promoters.

### Hemin reduces promoter-associated H3K4me3 and H3K27ac through decreased recruitment of histone-modifying enzymes

**Fig. 5:**
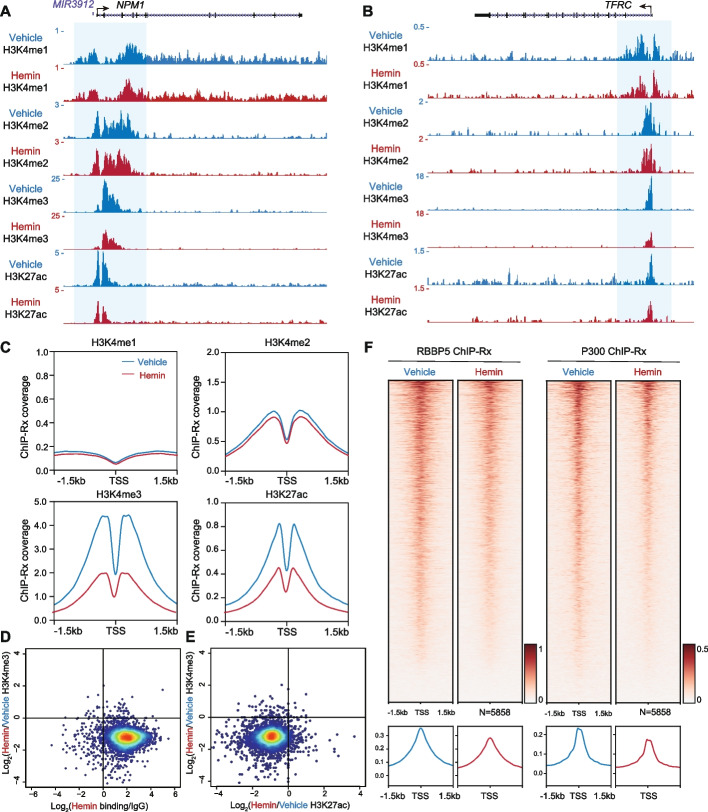
Hemin alters chromatin modifications at promoters with decreased H3K27ac and H3K4me3 levels. **A–C** H3K4me1, H3K4me2, H3K4me3, and H3K27ac ChIP-Rx signals in HEK293T cells treated with or without hemin. Track examples at the *NPM1* (**A**) and *TFRC* (**B**) loci are shown. As shown in metaplots (**C**), hemin treatment globally reduced H3K4me3 and H3K27ac levels at hemin-bound promoters. **D**, **E** Scatterplots of the log_2_ fold changes of H3K4me3 signals versus hemin binding signals (**D**) or the log_2_ fold changes of H3K27ac signals (**E**) after hemin treatment. The majority of hemin-bound promoters showed decreased H3K4me3 signals (**D**), and most of them were accompanied by decreased H3K27ac signals (**E**). **F** Heatmap and metaplot analyses of RBBP5 and P300 ChIP-Rx signals in the presence or absence of hemin. Hemin treatment impairs RBBP5 and P300 occupancy at hemin-bound promoters (*N*=5858)

Given the evidence that G4 marks transcriptional regulatory elements enriched with H3K4me1, H3K4me2, H3K4me3, and H3K27ac modifications [21, 22], we further investigated hemin-induced changes of these modifications in HEK293T cells with or without hemin treatment for 2 h. UCSC genome browser snapshots of ChIP-Rx illustrate that hemin treatment dramatically reduced H3K4me3 and H3K27ac levels at *NPM1* and *TFRC* promoters, while H3K4me1 and H3K4me2 levels were slightly decreased (Fig. 5A,B). In agreement with the distribution of G4s at the nucleosome-free regions of promoters [21], metaplot analysis showed that hemin induced a global decrease of H3K4me3 and H3K27ac, as well as a slight reduction of H3K4me1 and H3K4me2 at the 5858 promoters with hemin binding (Fig. 5C). Scatterplots confirmed that hemin treatment led to decreases in both H3K4me3 and H3K27ac at most hemin-bound promoters (Fig. 5D,E).

To determine if the observed decrease of H3K4me3 and H3K27ac is caused by reduced recruitment of histone-modifying enzymes, we performed ChIP-Rx with antibodies against the RB Binding Protein 5 (RBBP5) and the E1A Binding Protein P300 (EP300, P300). RBBP5 is a shared subunit for the COMPASS family of histone H3K4 methylases [63, 64] and P300 is a histone H3K27 acetyltransferase that regulates transcription via chromatin remodeling [65, 66]. Heatmap and metaplot analyses demonstrate that 2-h hemin treatment impairs RBBP5 and P300 occupancy at hemin-bound promoters (*N*=5858) (Fig. 5F), thus revealing a potential mechanism underlying the alteration of chromatin landscapes by hemin.

### G4 status is not involved in the hemin-BACH1-NRF2-mediated enhancer activation process

**Fig. 6:**
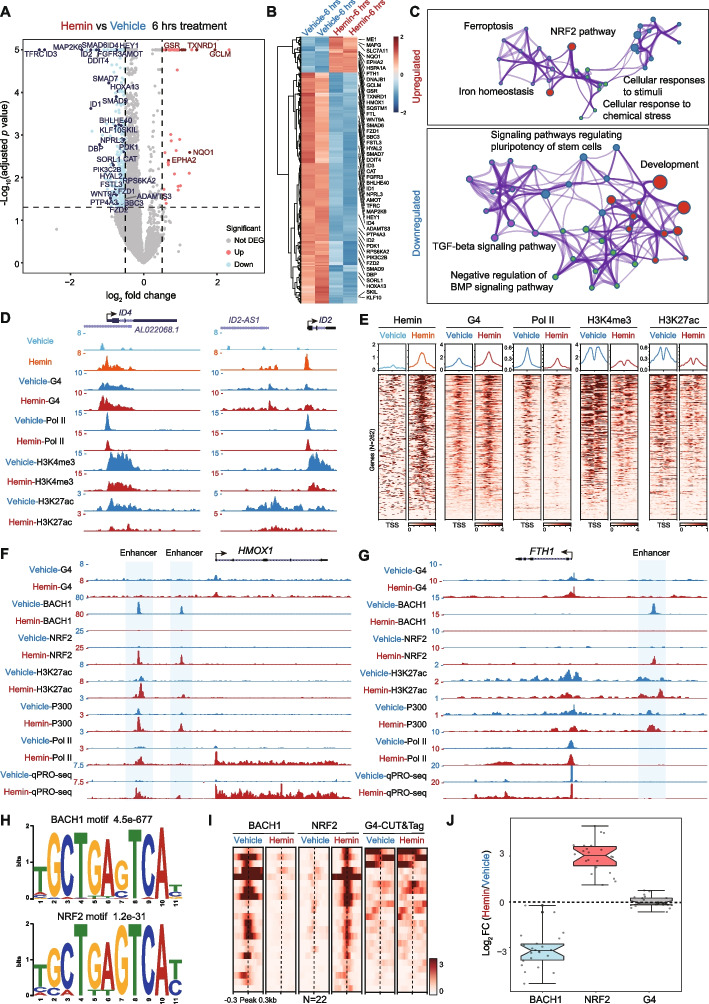
G4 status is not involved in the hemin-BACH1-NRF2-mediated enhancer activation process. **A, B** Volcano plot (**A**) and heatmap (**B**) showing gene expression changes in HEK293T cells in response to hemin treatment for 6 h. **C** Network enrichment analysis with Metascape [67] of upregulated and downregulated genes by hemin. Each cluster is represented by different colors and each enriched term is denoted by a circle node. **D**, **E** Analysis of hemin binding signals, G4-CUT&Tag signals, Pol II, H3K4me3, and H3K27ac ChIP-Rx signals at promoters of the downregulated genes by hemin (*N*=262). Track examples at the *ID4* and *ID2* loci (**D**), heatmaps and metaplots (**E**) with 2-kb windows are shown. Hemin treatment induced G4 formation, reduced Pol II occupancy, and altered Histone 3 modifications at hemin-bound promoters, which eventually perturbed gene expression. **F**, **G** UCSC genome browser snapshots of G4-CUT&Tag signals, BACH1, NRF2, H3K27ac, P300, and Pol II ChIP-Rx signals and qPRO-seq signals at NRF2-induced *HMOX1* (**F**) and *FTH1* (**G**) loci. Hemin-induced *HMOX1* and *FTH1* expression was mediated by the release of BACH1 and binding of NRF2 at enhancer regions, which were accompanied by increased P300 occupancy and H3K27ac level. Interestingly, G4 is not involved in the enhancer activation process. **H** Motif analysis of BACH1 and NRF2 binding sites. **I**, **J** Heatmap (**I**) and boxplot (**J**) analyses demonstrate that hemin induced BACH1 release and NRF2 binding, without major alteration of G4-CUT&Tag signals

Since hemin treatment impairs transcription initiation and modulates chromatin landscapes in HEK293T, to characterize the effects of hemin on gene expression and identify specific genes sensitive to hemin, we measured the mRNA levels after a relatively short period (6 h) of treatment in HEK293T cells, with or without hemin, using mRNA-seq with exogenous *Drosophila S2* cell spike-in RNA. We found that hemin-bound genes were generally associated with downregulation by hemin treatment (Additional file 1: Fig. S4A). Statistical analysis revealed that 46 genes were upregulated (fold change > 1.5 and adjusted *p* < 0.05), and 295 genes were downregulated (fold change > 1.5 and adjusted *p* < 0.05) significantly after a short period of hemin treatment (Fig. 6A,B). The 46 hemin-upregulated genes include known hemin-responsive genes related to the canonical hemin-BACH1-NRF2 pathway [41, 42]. These genes are enriched in ferroptosis, the NRF2 pathway, ion homeostasis, and cellular response to stimuli (Fig. 6C). Interestingly, gene ontology analysis of the 295 downregulated genes identified the TGF-beta signaling pathway, negative regulation of BMP signaling, development, and signaling pathways regulating pluripotency of stem cells, as significantly enriched categories, suggesting that hemin may play important roles in the TGF-beta family signaling pathway and other aspects of development as well. We further inspected these downregulated genes (Fig. 6D) and found that these downregulated genes contained hemin binding sites at their promoter regions and that hemin treatment could induce G4 formation, inhibit Pol II occupancy, and reduce H3K4me3 and H3K27ac levels at their promoter regions (Fig. 6E).

To determine if G4 is involved in the canonical hemin-BACH1-NRF2-mediated gene-regulatory pathway [39–42], we examined two well-known hemin-induced genes *HMOX1* and *FTH1*. We found that hemin treatment reduced BACH1 binding and increased NRF2 occupancy at nearby enhancers and led to enhancer activation (Fig. 6F,G). UCSC tracks of BACH1 and NRF2 at two well-known loci (*NQO1* and *FTL*) confirmed the specificity of BACH1 and NRF2 ChIP-Rx signals (Additional File 1: Fig. S4B). Motif analysis further confirmed the enrichment of antioxidant response elements at BACH1 and NRF2 binding peaks (Fig. 6H). Surprisingly, G4s were absent from the *HMOX1* and *FTH1* enhancers, and hemin treatment did not change the G4-CUT&Tag signals at these enhancers (Fig. 6F,G), indicating that G4 did not participate in the enhancer activation of *HMOX1* and *FTH1* genes. To investigate the generality of these findings, we performed a genome-wide analysis of all NRF2 binding sites and showed that BACH1 occupancy decreased at the NRF2 binding sites after hemin treatment (Fig. 6I,J). Consistent with the track examples (Fig. 6F,G), the G4-CUT&Tag signals at the NRF2 binding sites were not globally changed by hemin treatment (Fig. 6J), demonstrating that alternation of G4 status is not involved in hemin-BACH1-NRF2-mediated enhancer activation.

To investigate the effects of hemin treatment on gene expression, we analyzed the effect of hemin treatment on the set of hemin-bound enhancers (Additional file 1: Fig. S4C). We found that hemin treatment decreased enhancer activity as revealed by reduced qPRO-seq signals, as well as reduced Pol II and H3K27ac occupancy (Additional file 1: Fig. S4C), which is similar to our findings at the hemin-bound promoters (Fig. 4 and Fig. 5), and distinct from NRF2-activated enhancers (Fig. 6F,G). Together, these results show two distinct mechanisms underlying hemin-induced transcriptional regulation: the canonical hemin-BACH1-NRF2 pathway [41, 42] that activates hemin-targeted gene expression independent of G4, and a hemin-dependent G4 formation that impairs Pol II transcription initiation and decreases enhancer activity for gene repression.

### Hemin-induced gene expression profiling in mouse primary hepatocytes

**Fig. 7:**
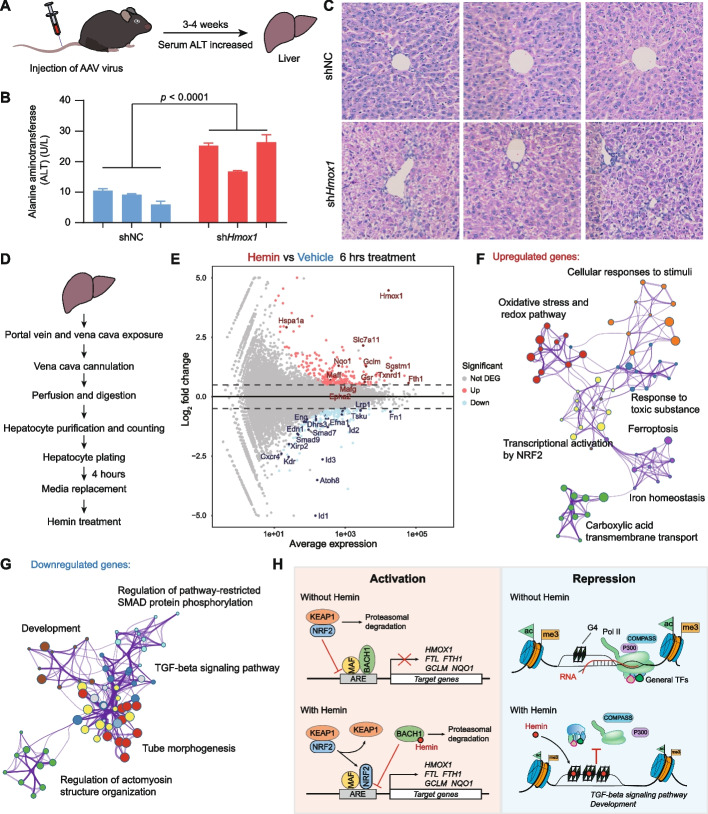
Hemin-induced gene expression profiling in mouse primary hepatocytes. **A** Eight-week-old C57BL/6 mice were injected with the recombinant AAV8-shNC or AAV8-sh*Hmox1* through tail vein injection. The mice were monitored with the serum alanine aminotransferase (ALT), and the mice with increased ALT levels were euthanized for further examinations. **B** Serum ALT levels of mice before they were euthanized for liver dissection. *Hmox1* knockdown induced serum ALT elevation, which indicated liver damage. **C** Hematoxylin and eosin (H&E) staining of the dissected liver tissues showing pathological changes in *Hmox1* knockdown mice. **D** Workflow for isolation and culture of mouse primary hepatocytes [69, 70]. **E** MA plot showing gene expression changes in mouse primary hepatocytes in response to hemin treatment for 6 h. **F,G** Network enrichment analysis of upregulated (**F**) and downregulated (**G**) genes by hemin treatment. Each cluster is represented by different colors and each enriched term is represented by a circle node. **H** Proposed model depicting two ways for hemin to participate in gene transcription regulation. In the BACH1-NRF2-dependent activation pathway, hemin induces degradation of BACH1 and binding of NRF2 to enhancer regions, which activates the transcription of target genes, including *HMOX1* and *FTH1*. In this study, we present a G4-dependent model for metabolic regulation of gene expression and chromatin landscapes by porphyrin metabolites. G4 works as a hemin sensor and could be stabilized by hemin. Hemin-induced G4 stabilization hampers the loading of Pol II, TFIIB, COMPASS, and P300, leading to the impairment of transcription initiation of target genes and the alteration of chromatin modifications

To study the consequences of hemin dysregulation in vivo, we used adeno-associated virus (AAV) to knockdown the hemin-degrading enzyme *Hmox1* in mouse liver, which also has a central role in porphyrin metabolism [68]. We first confirmed the knockdown efficiency of the sh*Hmox1* AAV virus using MEF cells (Additional file 1: Fig. S5A-B; Additional file 2) and injected the AAV virus into mice through tail veins (Fig. 7A). Three weeks after injection, the mice showed elevated serum alanine aminotransferase (ALT) activities (Fig. 7B), which is a typical sign of liver injury. Liver sections visualized using hematoxylin and eosin (H&E) staining showed hepatic damage with no obvious signs of immune cell infiltration (Fig. 7C). Analysis of dissected liver tissues confirmed the knockdown of *Hmox1* mRNA and an elevated concentration of hemin (Additional file 1: Fig. S5C-D). Additionally, we noticed that the hemin levels changed dynamically after the sh*Hmox1* AAV virus injection, which may be caused by a highly dynamic hemin metabolism or the reciprocal regulation between *Hmox1* and hemin in vivo [37, 38].

To minimize these potential mechanisms of interference in vivo, we isolated the mouse primary hepatocytes and cultured them ex vivo for hemin treatment (Fig. 7D). Treatment of mouse primary hepatocytes with hemin increased G4 formation (Additional file 1: Fig. S5E), which was consistent with our findings in HEK293T and HeLa cells (Fig. 3). mRNA-seq analysis was performed with primary hepatocytes after hemin treatment for 6 h (Fig. 7E; Additional file 1: Fig. S5F). Similar with the upregulated genes by hemin treatment in HEK293T cells, hemin treatment in primary hepatocytes led to upregulation of 258 genes which are enriched for processes related to transcriptional activation by NRF2, oxidative stress and redox pathways, iron homeostasis, and ferroptosis (Fig. 7F; Additional file 1: Fig. S5G). Of note, gene ontology analysis of hemin-downregulated genes revealed that the TGF-beta signaling pathway, regulation of cellular response to TGF-beta, and development were highly enriched terms (Fig. 7G; Additional file 1: Fig. S5G). These findings are very similar to our findings with human HEK293T cells (Fig. 6A–C), highlighting a potential regulation of TGF-beta family signaling pathways and potentially other aspects of development by hemin in mammals.

To explore the preference of hemin on TGF-beta family signaling pathways, we investigated whether the downregulated genes were related to the factors such as gene length, G4 levels, hemin binding intensities, and RNA half-lives [71]. We found that the downregulated genes did not have apparent bias for gene length (Additional file 1: Fig. S5H); however, they were related to higher G4 occupancy and hemin binding intensities at promoters (Additional file 1: Fig. S5I-J). Interestingly, we noticed that the downregulated genes in TGF-beta family signaling pathways, such as *ID1*, *ID2*, *ID3*, *ID4*, *SMAD6*, and *SMAD7*, were associated with shorter mRNA half-lives (Additional file 1: Fig. S5K), suggesting that post-transcriptional regulation of these transcripts may contribute to the observed preference of hemin treatment on TGF-beta family signaling pathways. Collectively, these data suggest that G4-dependent transcriptional repression through hemin is a well-conserved regulator of TGF-beta family signaling pathways and development.

## Discussion

Our studies propose that hemin regulates gene expression through two distinct mechanisms depending on the involvement of G-quadruplexes (Fig. 7H). First, hemin can induce BACH1 degradation and promote NRF2 binding at enhancers of antioxidant response elements for the activation of target genes related to ferroptosis and iron homeostasis (Fig. 7H). This enhancer activation process is mediated by the dynamic regulation of transcription factors at enhancers [39–42] and does not require the involvement of G4s. Second, in this study, we present a novel G4-dependent mechanism for metabolic regulation of gene expression and chromatin landscapes by natural porphyrin metabolites. We propose that G4 status can function as a sensor for hemin in the genome (Fig. 2), with increased hemin levels promoting G4 formation (Fig. 3). Through this mechanism, hemin could inhibit transcription initiation at promoters (Fig. 4) and alter chromatin landscapes with reduced histone H3K4me3 and H3K27ac levels (Fig. 5). Hemin treatment decreased the firefly luciferase reporter gene activities using recombinant plasmids inserted with *NPM1*, *HSPA8*, *H2AX*, *SERBP1*, or *SRSF4* promoters (Fig. 4G–I). However, it should be noted that this reporter gene assay may not fully recapitulate the normal transcriptional regulation mechanisms by G4 due to lacking of the chromatin context and high copy numbers of templates in each cells. In summary, these effects are distinct from the canonical hemin-BACH1-NRF2 pathway [41, 42] and, instead, directly contribute to the repression of target genes associated with TGF-beta family signaling pathways and development (Fig. 7H).

G4 can adopt different topologies including parallel, antiparallel, or hybrid (a mix of both) [30, 31]. Based on the intracellular distribution of natural porphyrin metabolites and their physical interaction with G4s (Fig. 1), we established an in situ capture sequencing method combining biotin-PEG4-hemin and the CUT&Tag approach, and mapped sites that can be bound by hemin in the context of chromatin (Fig. 2). Furthermore, we developed an assay to profile the hemin binding sites in the chromatin using the G4-hemin self-biotinylation reaction in combination with the CUT&Tag method (Fig. 2). We found that cellular hemin binding sites were distributed among promoter and intergenic regions and overlapped with a proportion of G4 peaks (Fig. 2H), suggesting that G4s act as sensors for hemin in cells, revealing a selectivity of hemin on G4 binding. Hemin preferentially binds to parallel MYC-G4 rather than antiparallel, mixed, and intermolecular G4s in vitro (Additional file 1: Fig. S1), which may be caused by the more accessible ends of parallel G4s [72, 73]. It will be very interesting to study the effects of hemin on RNA quadruplexes since they are typically parallel. However, it is not currently possible to characterize the topologies of G4s in cells, a worthy area of future investigation, with clear implications for the development of topological-specific quadruplex-targeting drugs.

Interestingly, hemin-induced modulation of enhancer activity depends on the status of BACH1 degradation, NRF2 binding, or G4 formation. Hemin activates the NRF2-occupied enhancers containing antioxidant response elements independent of G4, while hemin decreases the activities of G4-containing enhancers with decreased qPRO-seq signals, as well as Pol II and H3K27ac occupancy (Additional file 1: Fig. S4C), which were similar to the hemin-bound promoters (Figs. 4 and 5). Mechanistically, this could be mediated through impaired loading of general or sequence-specific transcription factors by hemin binding at G4s as well as epigenetic and conformational changes at promoters or enhancers [21]. Given the importance of enhancers in human diseases and development [74], it is tempting to explore the roles and selectivity of porphyrins in enhancer regulation.

Porphyrins are essential metabolites participating in a variety of biological processes such as oxidation, oxygen transportation, and electron transport. Although mounting evidence shows that cellular metabolites shape the structure of chromatin and directly regulate the transcriptional processes, the proposed mechanisms mainly focus on the binding of specialized histones, the key substrates of chromatin-modifying enzymes, and the activity of transcription factors and corepressors [8, 10]. Here, we present an additional mechanism of metabolic regulation of gene expression and chromatin modification through regulation of the formation of G4 nucleic acid structures.

PpIX in cells exists mainly in its free form, and the amounts of iron-bound PpIX are much lower than free PpIX (Fig. 1H,I). We found that treatment with PpIX increased G4 formation, which may be caused by its direct interaction with G4 (Fig. 1K), or enhanced conversion of PpIX to hemin (Fig. 3A–D). We found that treatment of cells with the precursor of porphyrins, ALA, resulted in increased G4 formation, which could be blocked by the ALAD enzyme inhibitor succinylacetone (Additional file 1: Fig. S3D-E), suggesting that porphyrin synthesis pathways have crucial roles in G4-dependent transcriptional regulation. Through mRNA-seq analysis, we found that hemin can rapidly repress the expression of specific genes in both HEK293T cells and mouse primary hepatocytes (Figs. 6 and 7), highlighting a potential role of hemin in regulating signaling pathways and development.

As a consequence of porphyrin dysregulation in vivo, reduction of the hemin-degrading enzyme *Hmox1* in mice liver resulted in hepatic damage (Fig. 7). Treatment of primary hepatocytes with hemin promoted G4 formation and induced specific gene expression profiles with downregulation of TGF-beta family signaling pathway genes and development-related genes (Fig. 7). The downregulated TGF-beta family signaling pathway genes are associated with higher G4 levels, higher hemin binding intensities, and shorter mRNA half-lives (Additional file 1: Fig. S4H-K), indicating that post-transcriptional regulation of these genes may contribute to the specificity of hemin-induced gene expression profiling. Of note, the TGF-beta family signaling pathway is a crucial pathway participating in all stages of liver disease progression, contributing to fibrosis, and promoting liver disease [75, 76]. Our results suggest that porphyrin dynamics may modulate liver function through regulation of the TGF-beta family signaling pathways.

## Conclusions

Our findings highlight an essential role of G4 in the recognition of porphyrins in cells and propose a model for metabolic regulation of gene expression and epigenetic landscapes through G4. Independent of the canonical hemin-BACH1-NRF2 activation of gene expression [41, 42], hemin promotes G4 formation genome-wide, inhibits transcription initiation, and decreases histone modifications at hemin-bound promoters, revealing an unprecedented role of porphyrin metabolites in the modulation of chromatin landscapes and gene transcription. These findings deepen our knowledge of G4 biology by providing novel biochemical mechanisms of G4 regulation by cellular metabolites, while more generally providing further insight into the interface of metabolic regulation and chromatin landscapes.

## Methods

### Cell lines

HEK293T, HeLa, U937, and K562 cells were obtained from ATCC. HEK293T and HeLa were cultured in DMEM (Gibco) supplemented with 10% fetal bovine serum (FBS, LONSERA). U937 and K562 cells were cultured in RPMI-1640 medium (Gibco) with 10% FBS. MDA-231-LM2 (LM2) cells were kindly provided by Dr. Yibin Kang and grown in DMEM supplemented with 10% FBS. *Drosophila S2* cells were obtained from Invitrogen (R690-07) and maintained in Schneider’s medium. Mouse embryonic fibroblast (MEF) cells were grown in DMEM with 10% FBS. All mammalian cells were cultured at 37°C with 5% CO_2_. Cells were confirmed to be mycoplasma negative using MycoBlue Mycoplasma Detector (Vazyme, D101-01). Live cells were quantified using a TC20 automated cell counter (Bio-Rad).

### Chemicals and antibodies

δ-Aminolevulinic acid hydrochloride (ALA; CAS: 5451-09-2), hemin (CAS: 16009-13-5), protoporphyrin IX (PpIX; CAS: 553-12-8), biotinyl tyramide (CAS: 41994-02-9), and succinylacetone (SA; CAS: 51568-18-4) were obtained from MedChemExpress (MCE) and dissolved in H_2_O, 0.1 M NaOH, and dimethyl sulfoxide (DMSO; Sigma-Aldrich), respectively, to make stock solution. Pyridostatin trihydrochloride (PDS; CAS: T4457) was purchased from Topscience Co., Ltd. Pol II Rpb1 (POLR2A) NTD (D8L4Y) rabbit mAb, Acetyl-Histone H3 (Lys27) (D5E4) XP Rabbit mAb, and TFIIB (GTF2B, 2F6A3H4) mouse mAb were purchased from Cell Signaling Technology. TBP mouse mAb (66166-1-Ig), BACH1 polyclonal antibody (14018-1-AP), and NRF2/NFE2L2 polyclonal antibody (16396-1-AP) were purchased from Proteintech. FLAG-synthetic antibody (M2) was obtained from Sigma-Aldrich. Biotin rabbit mAb (A20684), EP300 rabbit pAb (A13016), RBBP5 rabbit pAb (A6965), and mouse anti-His tag antibodies (AE003) were purchased from ABclonal. HMOX1 Rabbit pAb (380753) was obtained from Zen-bioscience. Homemade histone H3 Lysine 4 methylation antibodies were generated as previously described [77]. The scFv antibodies BG4 and BG4-EGFP were purified using the pSANG10 expression vector according to the published protocols [21, 56]. The specificity of purified BG4 and BG4-EGFP to G4 has been confirmed in a previous study [21]. Recombinant pA-Tn5 purification and pA-Tn5 transposome assembly were performed as described previously [21].

### Hemin and PpIX extraction from cells and analysis with LC-ESI-MS/MS

Hemin and PpIX were extracted from cells according to a previous study [52] with some modifications. Briefly, twenty million cells were collected, resuspended with 300 μL of TE buffer (10 mM Tris-HCl, 1mM EDTA at pH 7.2), and shaken for 1 h at room temperature. Samples were pre-chilled on ice and cells were efficiently disrupted with ultrasonication. Nine hundred microliters of acetonitrile was added to the sample with a 5-min incubation on a shaker at room temperature. After centrifugation at 2500*g* for 5 min, the supernatant-containing free porphyrin fraction was collected and analyzed for the amount of free PpIX and hemin with liquid chromatography with electrospray ionization tandem mass spectrometry **(**LC-ESI-MS/MS). Next, 800 μL of acetonitrile: 1.7 M HCl (8:2, *v*/*v*) was added to the pellet and shaken for 40 min at room temperature to extract PpIX and heme from the protein and chromatin. Two hundred microliters of saturated MgSO_4_ and 0.1 g of NaCl were added, and shaken for another 5 min. After centrifugation at 2500*g* for 5 min, the top organic layer was collected to analyze the content of protein-bound and chromatin-bound PpIX and hemin with LC-ESI-MS/MS.

LC-ESI-MS/MS experiments were performed on a Thermo TSQ Quantis triple-stage quadrupole mass spectrometer under the positive ion mode. Injections were automatically performed using an UltiMate 3000 HPLC equipped with an autosampler. A Hypersil GOLD C18 HPLC column was used for chromatographic separation at 40°C. The electrospray ionization was set at 3.5 kV, and the vaporizer temperature was set at 275°C. Selected reaction monitoring (SRM) mode was used, and the cycle time is 0.3 s. The mobile phase was composed of 0.1% formic acid H_2_O (A) and acetonitrile (B). The gradient condition was used: 0–0.5 min, 5% B; 0.5–5 min, 5–95% B; 5–7 min, 95% B; 7–8.1 min 95–5% B, 8.1–12 min 5% B. The flow rate was 0.2 mL/min. A volume of 5–10 μL of each sample was loaded for each analysis. The precursor m/z for PpIX was 564.2 and the product m/z were 432.28, 490.22, and 505.19 with collision energies of 50.7, 41.86, and 40.93 eV, respectively. Hemin has a precursor m/z of 616 and product m/z of 483.18, 498.14, and 557.21 with collision energies of 55, 52.05, and 38.82 eV.

### Bio-layer interferometry assay

Bio-layer interferometry assays were performed using a GatorPrime with Small Molecule, Antibody, and Protein (SMAP) Probes (Gator Bio, 160011), which are high-capacity streptavidin biosensors. Biotin-labeled ssDNA oligonucleotide (5′-[Biotin] GGC ATA GTG CGT GGG CG-3′) and its reverse complement DNA oligo were synthesized by Sangon Biotech and annealed to form biotin-labeled single-stranded and double-stranded DNA. Biotin-labeled MYC-G4 oligonucleotide (5′-[Biotin] TGA GGG TGG GTA GGG TGG GTA A-3′), SPB1-G4 oligonucleotide (5′-[Biotin] GGC GAG GGG CGT GGC CGG C-3′), hTELO-G4 oligonucleotide (5′-[Biotin] GGT TAG GGT TAG GGT TAG GGT TAG GGT TAG-3′), and intermolecular hTELO-G4 oligonucleotide (5′-[Biotin] TTA GGG TTA GGG TTA GGG-3′) were synthesized and annealed to form biotin-labeled parallel, antiparallel, mixed parallel/antiparallel, and intermolecular G4 structures. Kinetic titration series were performed in the interaction buffer (PBS with 0.5% DMSO and 0.02% Tween-20). PpIX and hemin were serially diluted with the interaction buffer from 20 to 1.25 μM and 8 to 0.5 μM, respectively. The SMAP biosensors were hydrated in the interaction buffer for 10 min at 25 °C. Following the initial 120-s baseline, the SMAP biosensors were loaded with the biotin-labeled G4 for 300 s. Redundant probes were removed by a 120-s baseline adjustment. To measure the interaction between porphyrins and G4, the duration time of association and dissociation were set to 120 s for PpIX and 300 s for hemin. One biosensor recorded the kinetic titration series by sequentially interacting with increasing concentrations of porphyrins, with dissociation between each sample concentration. Meanwhile, one sensor recorded the buffer reference signal and two sensors were used as sensor reference. Sensorgrams and sensor signals were analyzed by the Gator Part11 Software.

### Synthesis and purification of Biotin-PEG4-hemin

Synthesis and purification of biotin-PEG4-hemin were performed as previously reported [78] with minor modifications. Hemin and biotin-PEG4-hydrazide (CAS: 756525-97-0, Aladdin) were dissolved in DMF and DMSO at 4.4 and 68 mg/mL, respectively. A volume of 250 μL of hemin was mixed with 5 μL of biotin-PEG4-hydrazide and 1.4 mg of DCC (CAS: 538-75-0, Aladdin). Then the mixture was incubated on a shaker in the dark for 3 h at room temperature. Next, 5% pyridine (CAS: 110-86-1, Aladdin) was added to the sample and the mixture was applied to a CDS Empore C18 Extraction Disks. The biotin-PEG4-hemin was eluted with a gradient of 40–90% acetonitrile in the presence of 0.1% TFA. After vacuum freeze-drying, the sample was dissolved in a small amount of DMSO and stored at −80°C. LC-ESI-MS/MS was used to assay the synthesis of biotin-PEG4-hemin. The precursor m/z for biotin-PEG4-hemin was 1103.45, and the product m/z were 616.25 and 666.22 with collision energies of 55 eV for both.

### In situ profiling of hemin binding sites in the genome using biotin-PEG4-hemin-based capture sequencing

Biotin-PEG4-hemin was combined with the Tn5-based CUT&Tag strategy to identify hemin binding sites in the genome. Briefly, one million HEK293T or HeLa cells were resuspended in NE1 buffer (20 mM HEPES at pH 7.9, 10 mM KCl, 0.5 mM spermidine, 0.1% Triton X-100, 20% Glycerol, 1× protease inhibitors) and incubated on ice for 10 min. After centrifugation at 1300*g* for 10 min, nuclei were isolated and collected. These nuclei were washed once with PBS and resuspended with 100 μL wash buffer (20 mM HEPES at pH 7.5, 150 mM KCl, 0.5 mM spermidine, 1× protease inhibitors). Ten microliters pre-activated concanavalin A-coated magnetic beads (Smart-Lifesciences) were added to these nuclei and incubated on a rotator for 10 min at room temperature. The supernatant was removed, and bead-bound nuclei were resuspended in 100 μL dig-wash buffer (0.05% digitonin, 20 mM HEPES at pH 7.5, 150 mM KCl, 0.5 mM spermidine, 1× protease inhibitors) containing 2 mM EDTA. Biotin-PEG4-hemin was added to the sample to a final concentration of 3 μM and rotated overnight at 4°C in the dark. To verify the signal specificity, Biotin-PEG4-hemin was competed with 20 μM TMPyP4. These nuclei were washed three times in 200 μL dig-wash buffer to remove unbound biotin-PEG4-hemin. Next, anti-biotin rabbit mAb was diluted 100-fold in 100 μL dig-wash buffer with 2 mM EDTA and was added to the nuclei with 1-h rotation at room temperature. For the control sample, IgG was used to replace anti-biotin antibody. After washing once with dig-wash buffer, these nuclei were resuspended with 100 μL dig-wash buffer with 2 mM EDTA and 1 μL of mouse anti-rabbit IgG, incubated for another hour at room temperature, and further washed three times with dig-wash buffer. A 1:250 dilution of pA-Tn5 adapter complex (~0.2 μM) was prepared in 100 μL dig-300 buffer (0.05% digitonin, 20 mM HEPES at pH 7.5, 300 mM KCl, 0.5 mM spermidine, 1× protease inhibitors) and added to the nuclei followed by 1-h rotation at room temperature in the dark. After five washes with dig-300 buffer, the bead-bound nuclei were resuspended with 40 μL of tagmentation buffer (10 mM TAPS-NaOH at pH 8.5, 10 mM MgCl_2_, 7.5% DMF) and incubated at 37°C for 1 h. To terminate the tagmentation reaction, 1.5 μL of 0.5 M EDTA, 0.5 μL of 10% SDS, and 1 μL of 20 mg/mL proteinase K were added and incubated at 55°C for 1 h and then for 15 min at 75°C. The DNA was extracted with Sera-Mag carboxylate-modified magnetic beads (GE Healthcare) for library preparation. Twenty-one microliters DNA was mixed with a universal i5 and a uniquely barcoded i7 primer and amplified with NEB Q5 high-fidelity 2× master mix. The libraries were purified with 0.56–0.85× volume of Sera-Mag carboxylate-modified magnetic beads and subjected to Bioanalyzer DNA analysis and Illumina sequencing.

Reads were aligned to human (UCSC *hg38*) genome with Bowtie2 version 2.2.6 [79], using parameters: --local --very-sensitive --no-mixed --no-discordant --phred33 -I 10 -X 700. The aligned reads were normalized with total reads aligned (reads per million, r.p.m.). The track files were made with the bamCoverage command from deeptools 3.3.1 [80]. Peaks were called using the IgG controls and MACS2 version 2.1.2 [81] with default parameters and a *p*-value cutoff of 1E−7. The distribution of hemin binding peaks was annotated with ChIPpeakAnno. To perform the motif analysis, the summits of the called hemin binding peaks were extended to 50 bp to fetch the DNA sequences, and MEME-ChIP [82] was used. Heatmaps were made for the indicated windows using the average coverage (r.p.m.).

### Genome-wide mapping of endogenous G4-hemin complexes using a G4 self-biotinylation reaction

Identification of endogenous G4-hemin binding sites in the genome was performed using a previously reported self-biotinylation reaction [55] in combination with the Tn5-based CUT&Tag strategy. Briefly, HEK293T cells were treated with or without 80 μM Hemin for 2 h. To verify the signal specificity, hemin was displaced with 80 μM TMPyP4 or PDS [12] for another 2 h. The nuclei were isolated from one million cells and resuspended with 100 μL dig-wash buffer (0.05% digitonin, 20 mM HEPES at pH 7.5, 150 mM KCl, 0.5 mM spermidine, 1× protease inhibitors) containing 1 μL of 1 mg/mL RNase A and 3 mM biotin tyramide. Ten millimolars H_2_O_2_ was added, and the reaction was performed for 3 min on a shaker at room temperature [55]. The reaction was quenched with PBS solution containing 5 mM Trolox and 10 mM sodium ascorbate and washed with wash buffer (20 mM HEPES at pH 7.5, 150 mM KCl, 0.5 mM spermidine, 1× protease inhibitors). The nuclei were resuspended with 100 μL wash buffer and conjugated to 10 μL pre-activated concanavalin A-coated magnetic beads (Smart-Lifesciences). The bead-bound nuclei were washed three times with dig-wash buffer to remove redundant biotin tyramide and incubated with 50 μL dig-wash buffer containing 2 mM EDTA and 0.5μL of anti-biotin rabbit mAb overnight at 4°C. After washing once, these nuclei were resuspended with 100 μL dig-wash buffer with 2 mM EDTA and 1 μL of rabbit anti-mouse IgG and incubated for another hour at room temperature. The nuclei were further washed three times with dig-wash buffer and tagmented with the pA-Tn5 adapter complexes. The tagmented DNA was used for library preparation and Illumina sequencing. The CUT&Tag sequencing reads were aligned to the human genome (UCSC *hg38*) using Bowtie2 version 2.2.6 [68] with parameters: --local --very-sensitive --no-mixed --no-discordant --phred33 -I 10 -X 700. The reads were further normalized with total reads aligned (reads per million, r.p.m.). Peaks were called using the IgG controls and MACS2 version 2.1.2 [81] with default parameters and a *p*-value cutoff of 1E−7. Heatmaps were made for the indicated windows using the average coverage.

### G4-CUT&Tag and R-loop CUT&Tag

G4-CUT&Tag and R-loop CUT&Tag were performed as previously described [21, 61].

Briefly, one million cells were resuspended with NE1 buffer by gentle pipetting and incubated on ice for 10 min. The nuclei were isolated and conjugated to 10 μL pre-activated concanavalin A-coated magnetic beads (Smart-Lifesciences). The bead-bound nuclei were resuspended in 50 μL of dig-wash buffer and incubated with 5 μL 0.1 mg/mL BG4 primary antibody or 2 μg recombinant GST-His6-2×HBD protein. For the control samples, IgG was used to replace the BG4 antibody or GST-His6-2×HBD. After overnight incubation at 4°C, the liquid was removed and the bead-bound nuclei were washed once with dig-wash buffer. The Anti-Flag M2 or anti-HisTag antibody (1:100 dilution) were used to bind with BG4 primary antibody or GST-His6-2×HBD. The nuclei were washed with dig-wash buffer and the Rabbit anti-mouse IgG were used to bind with the Anti-Flag M2 or anti-HisTag antibody. Bead-bound nuclei were briefly washed three times with 200 μL of dig-wash buffer and tagmented with pA-Tn5 adapter complex for library preparation [21, 58].

G4-CUT&Tag and R-loop CUT&Tag reads were aligned to the human genome (UCSC *hg38*) and *Escherichia coli* genome with Bowtie2 version 2.2.6 [79], using parameters: --local --very-sensitive --no-mixed --no-discordant --phred33 -I 10 -X 700. The aligned reads were normalized with total reads aligned (reads per million, r.p.m.) and spiked-in *E. coli* reads. The track files were made with the bamCoverage command from deeptools 3.3.0 [80]. Peaks were called using MACS2 version 2.1.2 with default parameters [81], and a *p*-value cutoff of 1E−5. Heatmaps were made for the indicated windows using the average coverage.

### Chromatin immunoprecipitation sequencing with reference exogenous genome (ChIP-Rx)

ChIP-Rx was performed with 1×10^7^ human cells and 1×10^6^ MEF cells for spike-in normalization as described in a previous study [21]. For immunoprecipitation, sonicated chromatin was incubated with 10 μg of specific antibodies and 15 μL of pre-blocked Protein A/G beads (Smart-Lifesciences). After extensive washes, the captured DNA was eluted for library preparation using the NEBNext Ultra II DNA library prep kit for Illumina before sequencing on a NovaSeq 6000. ChIP-Rx reads were aligned to the human genome (UCSC *hg38*) and mouse genome (*mm10*) with Bowtie2 version 2.2.6 [79], using parameters: --local --very-sensitive. The resulting reads were normalized with the aligned mouse reads. The aligned human BAM files were normalized and converted to bigwig files for visualization in the UCSC Genome Browser. Peaks were called using MACS2 version 2.1.2 with default parameters [81]. MEME-ChIP [82] was used to further analyze peak region motifs. Heatmap and metaplots were made for the indicated windows using the average coverage (r.p.m.).

### Quick precision run-on sequencing (qPRO-seq)

qPRO-seq was performed as previously reported [21, 62], and qPRO-seq libraries were sequenced on the NovaSeq 6000 platform. qPRO-seq reads were aligned to the human *hg38* genome with Bowtie version 1.1.2 [83]. UMI-tools was used to extract UMIs and remove duplications [84]. The resulting reads were normalized to total reads used for alignment (reads per million, r.p.m.) and converted to bigwig files for visualization in the UCSC Genome Browser. Heatmap and metaplots were made for the indicated windows using the average coverage (r.p.m.).

### mRNA sequencing (mRNA-seq)

Following hemin treatment for 6 h, cells were collected and lysed with Trizol reagent. Total RNA was extracted according to the manufacturer’s instructions. One microgram total RNA was spike-in with 100 ng *Drosophila S2* RNA and was further used for mRNA isolation with VAHTS mRNA Capture Beads (Vazyme, N401) following the manufacturer’s instructions. Library preparation was performed with the NEBNext Ultra II Directional RNA Library Prep Kit, followed by sequencing on a NovaSeq 6000. RNA-seq reads were aligned to the human genome (UCSC *hg38*) or mouse genome (*mm10*). Alignments were processed with HISAT2 [85], allowing only uniquely mapping reads with up to three mismatches within the 150-bp reads. The read counts across each gene were counted with featureCounts version 2.0.0, and DESeq2 was used to perform the differential gene expression analysis [86]. To estimate the fold changes based on the *Drosophila* spike-in RNA, size factors were calculated on the counts of the *Drosophila* genes and applied to the human gene counts before fold change estimation with DESeq2. Heatmaps were generated using R package 3.4.3 with normalized counts from DESeq2. Gene ontology enrichment was analyzed with Metascape [67] for upregulated and downregulated genes in response to hemin treatment.

### Immunofluorescence microscopy and analysis

HeLa cells or mouse primary hepatocytes were seeded on a coverslip and cultured for 24 h before treatment. Cells were then fixed with pre-chilled methanol for 10 min. After three washes with PBS, cells were permeabilized with 0.1% Triton X-100 in PBS buffer for 15 min and blocked with 8% BSA in PBS for 1 h. Next, cells were incubated with 2 μg recombinant BG4-EGFP antibody in 2% BSA for 2 h and washed five times for 1 h with 0.1% Tween 20 in PBS under gentle rocking. For nuclear staining, cells were incubated with 2 μg/μL of DAPI (4',6-diamidino-2-phenylindole, dihydrochloride) (Thermo Fisher Scientific, D1306) for 15 min. After mounting, slides were imaged with a Leica SP8 microscope. EGFP was excited by the 488-nm laser line, and the fluorescence was detected in the range of 498–545 nm. The 405-nm laser line was used to excite DAPI, and the emission between 413 and 462 nm was collected. For each group, images of about 6 fields of vision were taken for every sample without Z-stack under fixed laser line intensity. For quantification of G4 intensities, the same number of nuclei in each group were randomly selected to calculate the mean fluorescence intensities of nuclear G4s by dividing the total intensities of BG4-EGFP with DAPI-marked nuclear area using the Leica LASX software. The same set of samples was imaged and processed in one batch to reduce experimental variation. The G4 staining profile of HeLa cells is available for download from figshare repository (10.6084/m9.figshare.21608112.v1 [87] and 10.6084/m9.figshare.21608100.v1 [57]).

### Reporter gene assays

Putative G4-forming regions in the promoters of *NPM1*, *SRSF4*, *HSPA8*, *H2AX*, and *SERBP1*, showing signal enrichments in G4-CUT&Tag, were inserted into the pGL3 basic luciferase reporter vector (Promega). HEK293T cells were seeded in 24-well plates and transfected with or without these recombinant plasmids. Two hours post-transfection, ALA, PpIX, or hemin were added to the medium. Then the cells were cultured for another 24 h before assaying for luciferase activity with the firefly luciferase assay kit (US Everbright Inc.) following the manufacturer’s instructions.

### Quantitative RT-PCR analysis

Cells were lysed with Trizol reagent, and total RNA was extracted according to the manufacturer’s instructions. Reverse transcription was performed using the ReverTra Ace qPCR RT Master Mix from Toyobo life science (FSQ-301). Real-time PCR was performed with ChamQ Universal SYBR qPCR Master Mix (Vazyme, Q711-02). Samples were amplified with primers for murine *Hmox1* gene (forward: 5′-AAG CCG AGA ATG CTG AGT TCA-3′; reverse: 5′-GCC GTG TAG ATA TGG TAC AAG GA-3′) and *18S rRNA* (forward: 5′-TGT GCC GCT AGA GGT GAA ATT-3′; reverse: 5′-TGG CAA ATG CTT TCG CTT T -3′) as a reference gene. The cycle threshold Ct values were normalized to the *18S rRNA* curve. PCR experiments were performed in triplicate and standard deviations were calculated and displayed as error bars.

### Isolation and culture of mouse primary hepatocytes

Isolation and culture of mouse primary hepatocytes were performed using a two-step perfusion method as previously published [69, 70] with some modifications. Eight-week-old C57BL/6 mice were used in this experiment. The anesthetized mice were placed on the dissection trap, and their limbs were secured using needles. A “U”-shaped incision was made through the skin, and both the portal vein and vena cava were exposed. Then the inferior vena cava was cannulated, and the liver was perfused with 20 mL of EGTA-containing buffer-I (2 mM glutamine, 0.5% glucose, 25 mM HEPES, 2 mM EGTA diluted in PBS) to wash out the blood and circulating cells from the liver as well as to eliminate calcium via EGTA. Next, 0.3 mg/mL collagenase type IV (Sigma-Aldrich) in Ca^2+^-containing buffer-II (2 mM glutamine, 0.5% glucose, 25 mM HEPES, 3 mM CaCl_2_ diluted in Williams E medium) was perfused to the liver to digest collagen in the extracellular matrix, thereby facilitating cell dispersion. The liver was gently dissected and transferred to a 10-cm plate. Collagenase-containing Ca^2+^-containing buffer-II was added to the plate, and the liver was ruptured by fine tip forceps and gentle pipetting. The suspension was filtered through a 70-μm cell strainer and ice-cold Ca^2+^-containing buffer-II was used to rinse the plate and added to the cell strainer. After centrifugation at 4°C, 50*g* for 3 min, the supernatant was removed and the hepatocyte-containing pellet was washed three times with ice-cold Ca^2+^-containing buffer-II. The isolated hepatocytes were resuspended with Williams E medium supplemented with 10% fetal bovine serum, 50 U/mL penicillin, 50 μg/mL streptomycin, 5 μg/mL insulin, and 50 μM hydrocortisone hemisuccinate. After counting living cells, these hepatocytes were plated on collagen-coated cell culture plates/wells. After being grown at 37°C with 5% CO_2_, the cells were rinsed with PBS and cultured in fresh medium overnight. The next day, cells were treated with hemin for immunofluorescence and RNA sequencing. The G4 staining profile used in our study is available for download from figshare (10.6084/m9.figshare.21608127.v1 [88]).

### Adeno-associated viral (AAV) production and transduction

The shRNA targeting murine *Hmox1* was constructed using the AAV-U6sgRNA (SapI)-hSyn-GFP-KASH-bGH vector (Addgene #60958). The sequence of sh*Hmox1* was 5′-ACC GAG CCA CAC AGC ACT ATG TAA ATT CAA GAG ATT TAC ATA GTG CTG TGT GGC TTT TTT TG-3′. For the production of recombinant AAV, HEK293T cells were seeded in 150-mm plates and co-transfected with pAAV-sh*Hmox1*, pAAV2/8-RC, and pHelper (1:1:1 molar ratio) using PEI MAX 40K (PolySciences). Seventy-two hours post-transfection, the cell culture supernatants were harvested, filtered with a 0.22-μm filter, and were further concentrated with Amicon Ultra-15 centrifugal filters (Millipore). The titer of recombinant AAV was determined by quantitative PCR as previously published [89]. To detect AAV knockdown efficiency, recombinant AAV viruses were used to transduce to the MEF cells. MEF cells were seeded in a 6-well plate and transduced by AAV with a multiplicity of infection (MOI) of 100,000 vgs per cell. Twenty-four hours post-transduction, cells were washed three times with PBS and cultured for another 48 h before collection. The cells were then analyzed by quantitative RT-PCR and western blot with an anti-HMOX1 antibody.

### Knockdown of Hmox1 by AAV in vivo

Eight-week-old C57BL/6 mice were injected with the recombinant AAV (5×10^11^ viral genome AAV8-shNC and AAV8-sh*Hmox1* diluted to 200 μL with PBS) through tail vein injection. Serum alanine aminotransferase (ALT) was monitored with the ALT assay kit (Nanjing Jiancheng Bioengineering Institute, C009) following the manufacturer’s instructions. Mice exhibiting increased serum ALT levels were euthanized, and their liver tissues were dissected for *Hmox1* RT-PCR analysis or hemin quantification. Collected mouse liver tissues were fixed with 4% neutral buffered formalin and embedded in paraffin. After deparaffinization, the sections were sequentially stained with the alum hematoxylin and eosin. Following dehydration and clearing, the sections were mounted in neutral balsam and imaged with Vectra 3 automated quantitative pathology imaging system (Akoya Biosciences). All animal experiments were conducted according to the guidelines of the ethics committee of the animal facility, Wuhan University. All mice were specified as pathogen-free and were housed under controlled temperature and light conditions following the animal care guidelines.

### Quantification and statistical analysis

Data are presented as Mean ± SD. The peak or gene size (*N*) in the heatmaps and scatter plots indicates the number of peaks or genes included. The sample sizes (*n*) in the figure legends indicate the number of replicates in each experiment. Statistical analyses in Fig. 3B–D, F–H, Figs. 4G–I, S3E, S5A, S5C, and S5D were performed by unpaired Student’s *t* test and the *p* values were denoted in each figure. The nested *t*-test was used for statistical analysis in Fig. 7B. Kolmogorov-Smirnov test (K-S test) was used for statistical test in Figures S3H and S3J.

## Supplementary Information

**Additional file 1: Figure S1**. Bio-layer interferometry analysis of hemin with oligonucleotides. **Figure S2**. Genome-wide profiling of hemin binding sites in HeLa cells using biotin-PEG4-hemin and capture sequencing. **Figure S3.** Effects of hemin on G-quadruplexes and R-loops at hemin binding sites. **Figure S4.** Decreased enhancer activation at hemin-bound enhancers. **Figure S5.** Effects of hemin on gene expression in mouse primary hepatocytes.

**Additional file 2: **Uncropped blot images.

**Additional file 3: **Review history.

## Data Availability

The raw and processed G4-CUT&Tag, ChIP-Rx, qPRO-seq, in situ biotin-PEG4-hemin CUT&Tag, G4 self-biotinylation, and mRNA-seq datasets supporting the conclusions of this article are available in the Gene Expression Omnibus (GEO) repository with accession number GSE198658 [90]. Publicly available G4-CUT&Tag data in HEK293T and HeLa used in this study were downloaded from the Gene Expression Omnibus (GEO): SRX11196891 and SRX11196903 [21]. The RNA half-life data from HEK293T cells were downloaded from GEO: GSE49831 [71]. Whole-genome sequencing data of HEK293T and HeLa were downloaded from the NCBI Sequence Read Archive with accession numbers SRX6858029 and ERX4517391 (SRA; https://www.ncbi.nlm.nih.gov/sra). All microscopy images are available at Figshare repository and can be accessed (10.6084/m9.figshare.21608112.v1 [87], 10.6084/m9.figshare.21608100.v1 [57], and 10.6084/m9.figshare.21608127.v1 [88]). All the other data generated in this study are included in the article and the Additional files.

## References

[1] Fuda NJ, Ardehali MB, Lis JT (2009). Defining mechanisms that regulate RNA polymerase II transcription in vivo. Nature.

[2] Roeder RG (2019). 50+ years of eukaryotic transcription: an expanding universe of factors and mechanisms. Nat Struct Mol Biol.

[3] Chen FX, Smith ER, Shilatifard A (2018). Born to run: control of transcription elongation by RNA polymerase II. Nat Rev Mol Cell Biol.

[4] Adelman K, Lis JT (2012). Promoter-proximal pausing of RNA polymerase II: emerging roles in metazoans. Nat Rev Genet.

[5] Zhao S, Allis CD, Wang GG (2021). The language of chromatin modification in human cancers. Nat Rev Cancer.

[6] van der Knaap JA, Verrijzer CP (2016). Undercover: gene control by metabolites and metabolic enzymes. Genes Dev.

[7] Haws SA, Yu D, Ye C, Wille CK, Nguyen LC, Krautkramer KA, Tomasiewicz JL, Yang SE, Miller BR, Liu WH (2020). Methyl-metabolite depletion elicits adaptive responses to support heterochromatin stability and epigenetic persistence. Mol Cell.

[8] Ladurner AG (2006). Rheostat control of gene expression by metabolites. Mol Cell.

[9] Li X, Egervari G, Wang Y, Berger SL, Lu Z (2018). Regulation of chromatin and gene expression by metabolic enzymes and metabolites. Nat Rev Mol Cell Biol.

[10] Gut P, Verdin E (2013). The nexus of chromatin regulation and intermediary metabolism. Nature.

[11] Mentch SJ, Mehrmohamadi M, Huang L, Liu X, Gupta D, Mattocks D, Gomez Padilla P, Ables G, Bamman MM, Thalacker-Mercer AE (2015). Histone methylation dynamics and gene regulation occur through the sensing of one-carbon metabolism. Cell Metab.

[12] Hansel-Hertsch R, Di Antonio M, Balasubramanian S (2017). DNA G-quadruplexes in the human genome: detection, functions and therapeutic potential. Nat Rev Mol Cell Biol.

[13] Varshney D, Spiegel J, Zyner K, Tannahill D, Balasubramanian S (2020). The regulation and functions of DNA and RNA G-quadruplexes. Nat Rev Mol Cell Biol.

[14] Marsico G, Chambers VS, Sahakyan AB, McCauley P, Boutell JM, Antonio MD, Balasubramanian S (2019). Whole genome experimental maps of DNA G-quadruplexes in multiple species. Nucleic Acids Res.

[15] Biffi G, Tannahill D, McCafferty J, Balasubramanian S (2013). Quantitative visualization of DNA G-quadruplex structures in human cells. Nat Chem.

[16] Zheng KW, Zhang JY, He YD, Gong JY, Wen CJ, Chen JN, Hao YH, Zhao Y, Tan Z (2020). Detection of genomic G-quadruplexes in living cells using a small artificial protein. Nucleic Acids Res.

[17] Spiegel J, Adhikari S, Balasubramanian S (2020). The structure and function of DNA G-Quadruplexes. Trends Chem.

[18] De Cian A, Cristofari G, Reichenbach P, De Lemos E, Monchaud D, Teulade-Fichou MP, Shin-Ya K, Lacroix L, Lingner J, Mergny JL (2007). Reevaluation of telomerase inhibition by quadruplex ligands and their mechanisms of action. Proc Natl Acad Sci U S A.

[19] Lyu J, Shao R, Kwong Yung PY, Elsasser SJ (2022). Genome-wide mapping of G-quadruplex structures with CUT&Tag. Nucleic Acids Res.

[20] Zyner KG, Simeone A, Flynn SM, Doyle C, Marsico G, Adhikari S, Portella G, Tannahill D, Balasubramanian S (2022). G-quadruplex DNA structures in human stem cells and differentiation. Nat Commun.

[21] Li C, Wang H, Yin Z, Fang P, Xiao R, Xiang Y, Wang W, Li Q, Huang B, Huang J, Liang K (2021). Ligand-induced native G-quadruplex stabilization impairs transcription initiation. Genome Res.

[22] Hansel-Hertsch R, Beraldi D, Lensing SV, Marsico G, Zyner K, Parry A, Di Antonio M, Pike J, Kimura H, Narita M (2016). G-quadruplex structures mark human regulatory chromatin. Nat Genet.

[23] Raiber EA, Kranaster R, Lam E, Nikan M, Balasubramanian S (2012). A non-canonical DNA structure is a binding motif for the transcription factor SP1 in vitro. Nucleic Acids Res.

[24] Gao J, Zybailov BL, Byrd AK, Griffin WC, Chib S, Mackintosh SG, Tackett AJ, Raney KD (2015). Yeast transcription co-activator Sub1 and its human homolog PC4 preferentially bind to G-quadruplex DNA. Chem Commun (Camb).

[25] Li PT, Wang ZF, Chu IT, Kuan YM, Li MH, Huang MC, Chiang PC, Chang TC, Chen CT (2017). Expression of the human telomerase reverse transcriptase gene is modulated by quadruplex formation in its first exon due to DNA methylation. J Biol Chem.

[26] Makowski MM, Grawe C, Foster BM, Nguyen NV, Bartke T, Vermeulen M (2018). Global profiling of protein-DNA and protein-nucleosome binding affinities using quantitative mass spectrometry. Nat Commun.

[27] Agarwal T, Roy S, Kumar S, Chakraborty TK, Maiti S (2014). In the sense of transcription regulation by G-quadruplexes: asymmetric effects in sense and antisense strands. Biochemistry.

[28] Belotserkovskii BP, Soo Shin JH, Hanawalt PC (2017). Strong transcription blockage mediated by R-loop formation within a G-rich homopurine-homopyrimidine sequence localized in the vicinity of the promoter. Nucleic Acids Res.

[29] Lee CY, McNerney C, Ma K, Zhao W, Wang A, Myong S (2020). R-loop induced G-quadruplex in non-template promotes transcription by successive R-loop formation. Nat Commun.

[30] Burge S, Parkinson GN, Hazel P, Todd AK, Neidle S (2006). Quadruplex DNA: sequence, topology and structure. Nucleic Acids Res.

[31] Miyoshi D, Karimata H, Sugimoto N (2006). Hydration regulates thermodynamics of G-quadruplex formation under molecular crowding conditions. J Am Chem Soc.

[32] Lago S, Nadai M, Cernilogar FM, Kazerani M, Dominiguez Moreno H, Schotta G, Richter SN (2021). Promoter G-quadruplexes and transcription factors cooperate to shape the cell type-specific transcriptome. Nat Commun.

[33] Rodriguez R, Miller KM, Forment JV, Bradshaw CR, Nikan M, Britton S, Oelschlaegel T, Xhemalce B, Balasubramanian S, Jackson SP (2012). Small-molecule-induced DNA damage identifies alternative DNA structures in human genes. Nat Chem Biol.

[34] Carvalho J, Mergny JL, Salgado GF, Queiroz JA, Cruz C (2020). G-quadruplex, Friend or Foe: The Role of the G-quartet in Anticancer Strategies. Trends Mol Med.

[35] Ruggiero E, Richter SN (2018). G-quadruplexes and G-quadruplex ligands: targets and tools in antiviral therapy. Nucleic Acids Res.

[36] Spiegel J, Cuesta SM, Adhikari S, Hansel-Hertsch R, Tannahill D, Balasubramanian S (2021). G-quadruplexes are transcription factor binding hubs in human chromatin. Genome Biol.

[37] Ponka P (1999). Cell biology of heme. Am J Med Sci.

[38] Ponka P, Sheftel AD, English AM, Scott Bohle D, Garcia-Santos D (2017). Do mammalian cells really need to export and import heme?. Trends Biochem Sci.

[39] DeNicola GM, Karreth FA, Humpton TJ, Gopinathan A, Wei C, Frese K, Mangal D, Yu KH, Yeo CJ, Calhoun ES (2011). Oncogene-induced Nrf2 transcription promotes ROS detoxification and tumorigenesis. Nature.

[40] Wiel C, Le Gal K, Ibrahim MX, Jahangir CA, Kashif M, Yao H, Ziegler DV, Xu X, Ghosh T, Mondal T (2019). BACH1 stabilization by antioxidants stimulates lung cancer metastasis. Cell.

[41] Sun J, Brand M, Zenke Y, Tashiro S, Groudine M, Igarashi K (2004). Heme regulates the dynamic exchange of Bach1 and NF-E2-related factors in the Maf transcription factor network. Proc Natl Acad Sci U S A.

[42] Lignitto L, LeBoeuf SE, Homer H, Jiang S, Askenazi M, Karakousi TR, Pass HI, Bhutkar AJ, Tsirigos A, Ueberheide B (2019). Nrf2 activation promotes lung cancer metastasis by inhibiting the degradation of Bach1. Cell.

[43] Liao R, Zheng Y, Liu X, Zhang Y, Seim G, Tanimura N, Wilson GM, Hematti P, Coon JJ, Fan J (2020). Discovering how heme controls genome function through heme-omics. Cell Rep.

[44] Tanimura N, Miller E, Igarashi K, Yang D, Burstyn JN, Dewey CN, Bresnick EH (2016). Mechanism governing heme synthesis reveals a GATA factor/heme circuit that controls differentiation. EMBO Rep.

[45] Travascio P, Witting PK, Mauk AG, Sen D (2001). The peroxidase activity of a hemin--DNA oligonucleotide complex: free radical damage to specific guanine bases of the DNA. J Am Chem Soc.

[46] Li Y, Sen D (1996). A catalytic DNA for porphyrin metallation. Nat Struct Biol.

[47] Gray LT, Puig Lombardi E, Verga D, Nicolas A, Teulade-Fichou MP, Londono-Vallejo A, Maizels N (2019). G-quadruplexes sequester free heme in living cells. Cell Chem Biol.

[48] Kaya-Okur HS, Wu SJ, Codomo CA, Pledger ES, Bryson TD, Henikoff JG, Ahmad K, Henikoff S (1930). CUT&Tag for efficient epigenomic profiling of small samples and single cells. Nat Commun.

[49] Wang Q, Xiong H, Ai S, Yu X, Liu Y, Zhang J, He A (2019). CoBATCH for high-throughput single-cell epigenomic profiling. Mol Cell.

[50] Gardner LC, Cox TM (1988). Biosynthesis of heme in immature erythroid cells. The regulatory step for heme formation in the human erythron. J Biol Chem.

[51] Hopp MT, Schmalohr BF, Kuhl T, Detzel MS, Wissbrock A, Imhof D (2020). Heme determination and quantification methods and their suitability for practical applications and everyday use. Anal Chem.

[52] Fyrestam J, Östman C (2017). Determination of heme in microorganisms using HPLC-MS/MS and cobalt (III) protoporphyrin IX inhibition of heme acquisition in Escherichia coli. Anal Bioanal Chem.

[53] Grand CL, Han H, Munoz RM, Weitman S, Von Hoff DD, Hurley LH, Bearss DJ (2002). The cationic porphyrin TMPyP4 down-regulates c-MYC and human telomerase reverse transcriptase expression and inhibits tumor growth in vivo. Mol Cancer Ther.

[54] Einarson OJ, Sen D (2017). Self-biotinylation of DNA G-quadruplexes via intrinsic peroxidase activity. Nucleic Acids Res.

[55] Lat PK, Liu K, Kumar DN, Wong KKL, Verheyen EM, Sen D (2020). High specificity and tight spatial restriction of self-biotinylation by DNA and RNA G-Quadruplexes complexed in vitro and in vivo with Heme. Nucleic Acids Res.

[56] Hansel-Hertsch R, Spiegel J, Marsico G, Tannahill D, Balasubramanian S (2018). Genome-wide mapping of endogenous G-quadruplex DNA structures by chromatin immunoprecipitation and high-throughput sequencing. Nat Protoc.

[57] Li C, Liang K: EGFP-BG4 staining of HeLa cells after Vehicle, PpIX or Hemin treatment. figshare; 2022. 10.6084/m9.figshare.21608100.v1.

[58] Wang H, Li C, Liang K (2022). Genome-wide native R-loop profiling by R-loop cleavage under targets and tagmentation (R-Loop CUT&Tag). Methods Mol Biol.

[59] Duquette ML, Handa P, Vincent JA, Taylor AF, Maizels N (2004). Intracellular transcription of G-rich DNAs induces formation of G-loops, novel structures containing G4 DNA. Genes Dev.

[60] De Magis A, Manzo SG, Russo M, Marinello J, Morigi R, Sordet O, Capranico G (2019). DNA damage and genome instability by G-quadruplex ligands are mediated by R loops in human cancer cells. Proc Natl Acad Sci U S A.

[61] Wang K, Wang H, Li C, Yin Z, Xiao R, Li Q, Xiang Y, Wang W, Huang J, Chen L (2021). Genomic profiling of native R loops with a DNA-RNA hybrid recognition sensor. Sci Adv.

[62] Judd J, Wojenski LA, Wainman LM, Tippens ND, Rice EJ, Dziubek A, et al. A rapid, sensitive, scalable method for Precision Run-On sequencing (PRO-seq). bioRxiv. 2020:2020.05.18.102277.

[63] Mohan M, Herz HM, Shilatifard A (2012). SnapShot: histone lysine methylase complexes. Cell.

[64] Shilatifard A (2012). The COMPASS family of histone H3K4 methylases: mechanisms of regulation in development and disease pathogenesis. Annu Rev Biochem.

[65] Delvecchio M, Gaucher J, Aguilar-Gurrieri C, Ortega E, Panne D (2013). Structure of the p300 catalytic core and implications for chromatin targeting and HAT regulation. Nat Struct Mol Biol.

[66] Ogryzko VV, Schiltz RL, Russanova V, Howard BH, Nakatani Y (1996). The transcriptional coactivators p300 and CBP are histone acetyltransferases. Cell.

[67] Tripathi S, Pohl MO, Zhou Y, Rodriguez-Frandsen A, Wang G, Stein DA, Moulton HM, DeJesus P, Che J, Mulder LC (2015). Meta- and orthogonal integration of influenza "OMICs" data defines a role for UBR4 in virus budding. Cell Host Microbe.

[68] Bloomer JR (1998). Liver metabolism of porphyrins and haem. J Gastroenterol Hepatol.

[69] Berry MN, Friend DS (1969). High-yield preparation of isolated rat liver parenchymal cells: a biochemical and fine structural study. J Cell Biol.

[70] Kudira R, Sharma BK, Mullen M, Mohanty SK, Donnelly B, Tiao GM, Miethke A (2021). Isolation and culturing primary chaolangiocytes from mouse liver. Bio Protoc.

[71] Schueler M, Munschauer M, Gregersen LH, Finzel A, Loewer A, Chen W, Landthaler M, Dieterich C (2014). Differential protein occupancy profiling of the mRNA transcriptome. Genome Biol.

[72] Cheng X, Liu X, Bing T, Cao Z, Shangguan D (2009). General peroxidase activity of G-quadruplex-hemin complexes and its application in ligand screening. Biochemistry.

[73] Kosman J, Juskowiak B (2011). Peroxidase-mimicking DNAzymes for biosensing applications: a review. Anal Chim Acta.

[74] Smith E, Shilatifard A (2014). Enhancer biology and enhanceropathies. Nat Struct Mol Biol.

[75] Kyritsi K, Kennedy L, Meadows V, Hargrove L, Demieville J, Pham L, Sybenga A, Kundu D, Cerritos K, Meng F (2021). Mast cells induce ductular reaction mimicking liver injury in mice through mast cell-derived transforming growth factor Beta 1 signaling. Hepatology.

[76] Fabregat I, Moreno-Caceres J, Sanchez A, Dooley S, Dewidar B, Giannelli G, Ten Dijke P, Consortium IL (2016). TGF-beta signalling and liver disease. FEBS J.

[77] Liang K, Woodfin AR, Slaughter BD, Unruh JR, Box AC, Rickels RA, Gao X, Haug JS, Jaspersen SL, Shilatifard A (2015). Mitotic transcriptional activation: clearance of actively engaged Pol II via transcriptional elongation control in mitosis. Mol Cell.

[78] Ishida M, Dohmae N, Shiro Y, Isogai Y (2003). Synthesis of biotinylated heme and its application to panning heme-binding proteins. Anal Biochem.

[79] Langmead B, Salzberg SL (2012). Fast gapped-read alignment with Bowtie 2. Nat Methods.

[80] Ramirez F, Ryan DP, Gruning B, Bhardwaj V, Kilpert F, Richter AS, Heyne S, Dundar F, Manke T (2016). deepTools2: a next generation web server for deep-sequencing data analysis. Nucleic Acids Res.

[81] Zhang Y, Liu T, Meyer CA, Eeckhoute J, Johnson DS, Bernstein BE, Nusbaum C, Myers RM, Brown M, Li W, Liu XS (2008). Model-based analysis of ChIP-Seq (MACS). Genome Biol.

[82] Machanick P, Bailey TL (2011). MEME-ChIP: motif analysis of large DNA datasets. Bioinformatics.

[83] Langmead B, Trapnell C, Pop M, Salzberg SL (2009). Ultrafast and memory-efficient alignment of short DNA sequences to the human genome. Genome Biol.

[84] Smith T, Heger A, Sudbery I (2017). UMI-tools: modeling sequencing errors in Unique Molecular Identifiers to improve quantification accuracy. Genome Res.

[85] Kim D, Paggi JM, Park C, Bennett C, Salzberg SL (2019). Graph-based genome alignment and genotyping with HISAT2 and HISAT-genotype. Nat Biotechnol.

[86] Love MI, Huber W, Anders S (2014). Moderated estimation of fold change and dispersion for RNA-seq data with DESeq2. Genome Biol.

[87] Li C, Liang K: EGFP-BG4 staining of HeLa cells after ALA treatment. figshare; 2022. 10.6084/m9.figshare.21608112.v1.

[88] Li C, Liang K: EGFP-BG4 staining of primary mouse hepatocytes. figshare; 2022. 10.6084/m9.figshare.21608127.v1.

[89] Teng Y, Xu Z, Zhao K, Zhong Y, Wang J, Zhao L, Zheng Z, Hou W, Zhu C, Chen X (2021). Novel function of SART1 in HNF4α transcriptional regulation contributes to its antiviral role during HBV infection. J Hepatol.

[90] Liang K. G-quadruplexes sense natural porphyrin metabolites for regulation of gene transcription and chromatin landscapes. Gene Expression Omnibus. 2022; https://https.ncbi.nlm.nih.gov/geo/query/acc.cgi?acc=GSE198658. 10.1186/s13059-022-02830-8 PMC9753424 36522639

