# Supplementary material for: G-quadruplexes sense natural porphyrin metabolites for regulation of gene transcription and chromatin landscapes
Source: Genome Biol. 2022 Dec 15;23:259. doi: 10.1186/s13059-022-02830-8 (PMC9753424; doi:10.1186/s13059-022-02830-8)
Supplement: Supplementary file 1 — Additional file 1: Figure S1. Bio-layer interferometry analysis of hemin with oligonucleotides. Figure S2. Genome-wide profiling of hemin binding sites in HeLa cells using biotin-PEG4-hemin and capture sequencing. Figure S3. Effects of hemin on G-quadruplexes and R-loops at hemin binding sites. Figure S4. Decreased enhancer activation at hemin-bound enhancers. Figure S5. Effects of hemin on gene expression in mouse primary hepatocytes. [file 13059_2022_2830_MOESM1_ESM.docx]

**G-quadruplexes sense natural porphyrin metabolites for regulation of gene transcription and chromatin landscapes**

Conghui Li ^1,†^, Zhinang Yin ^1,†^, Ruijing Xiao ^1,†^, Beili Huang ^1^, Yali Cui ^1^, Honghong Wang ^1^, Ying Xiang ^1^, Lingrui Wang ^1^, Lingyu Lei ^1^, Jiaqin Ye ^1^, Tianyu Li ^1^, Youquan Zhong ^1^, Fangteng Guo ^1^, Yuchen Xia ^1,2,3^, Pingping Fang ^1,^*, Kaiwei Liang ^1,3,4^*

^1^ School of Basic Medical Sciences, Wuhan University, Wuhan 430071, China

^2^ State Key Laboratory of Virology and Hubei Province Key Laboratory of Allergy and Immunology, Wuhan University, Wuhan 430071, China

^3^ TaiKang Center for Life and Medical Sciences, TaiKang Medical School, Wuhan University, Wuhan 430071, China

^4^ Hubei Province Key Laboratory of Allergy and Immunology, School of Basic Medical Sciences, Wuhan University, Wuhan 430071, China

^†^ contributed equally to this work.

^*^ To whom correspondence should be addressed


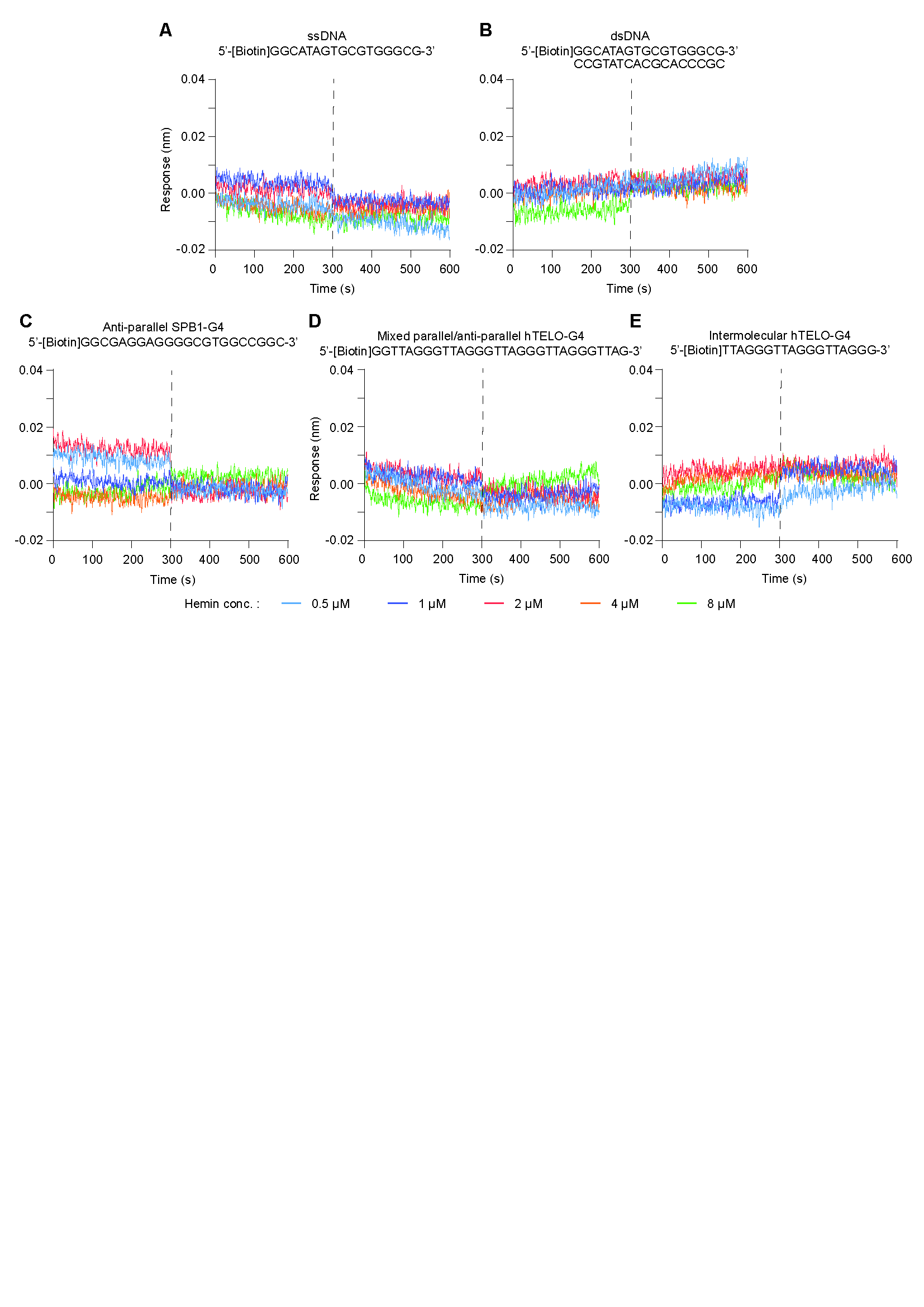


**Figure S1. Bio-layer interferometry analysis of hemin with oligonucleotides**

(**A and B**) Bio-layer interferometry analysis of hemin with single-stranded DNA (A) and double-stranded DNA (B). Biotinylated oligonucleotides were immobilized on streptavidin biosensors and incubated with a range of hemin concentrations to measure the response in a Gator instrument. (**C-E**) Characterization of the interaction between hemin and G4s. Hemin showed no binding with anti-parallel SPB1-G4 (C), mixed parallel/anti-parallel hTELO-G4 (D), or intermolecular hTELO-G4 (E)[15] at hemin concentrations from 0.5 μM to 8 μM.





**Figure S2. Genome-wide profiling of hemin binding sites in HeLa cells using biotin-PEG4-hemin and capture sequencing**

**(A)** Dot blot analysis of biotin-PEG4-hemin with single-stranded DNA, double-stranded DNA, and MYC-G4. DNA probes was spotted on the Hybond nylon membrane, and were incubated with 1 μM biotin-PEG4-hemin. After wash, the membrane was further incubated with anti-biotin antibody and HRP-conjugated secondary antibody before exposure. **(B)** Size distribution of biotin-PEG4-hemin CUT&Tag library. **(C)** Track examples of hemin binding sites and G4-CUT&Tag signals at the *NPM1* locus in HeLa cells. **(D)** Genome-wide annotation of hemin binding peaks in HeLa cells. **(E)** G4 prediction using hemin binding sequences in HeLa cells (upper panel) and random sequences (bottom panel) from the human genome by G4Hunter. 21,359 hemin binding peaks predicted 93,480 G4 motifs, while the matched random sequences generated only 12,459 hits. The distribution of absolute G4Hunter scores was shown in histogram plots. **(F and G)** Heatmap (F) and metaplot (G) analysis of hemin binding peaks and G4-CUT&Tag peaks in HeLa cells. 37.9% of G4-CUT&Tag peaks overlapped with hemin binding peaks. **(H)** Motif analysis of hemin binding sequences in HeLa cells showing the possibility of G4 formation.





**Figure S3. Effects of hemin on G-quadruplexes and R-loops at hemin binding sites**

**(A and B)** Analysis of the purified BG4 (~31 KD, arrow) (A) and BG4-EGFP (~61 KD, arrow) (B) proteins by SDS-PAGE and Coomassie blue staining. Non-specific products were indicated with asterisks. **(C)** Immunostaining of HeLa cells without BG4-EGFP served as negative controls. HeLa cells were treated with PpIX or hemin for 2 hours and the nuclei were stained with DAPI (blue). **(D and E)** Immunostaining of G4 with BG4-EGFP (green) in HeLa cells treated with 10 mM ALA and/or 100 μM succinylacetone (SA) for 6 hours. The nuclei were stained with DAPI (blue) (D). For each sample, images of 6 fields of vision were acquired with fixed parameters and 70 nuclei were randomly selected for quantification by dividing the total intensities of BG4-EGFP with DAPI marked the nuclear area (E) (https://doi.org/10.6084/m9.figshare.21608112.v1 [87]). **(F)** Scheme of R-loop CUT&Tag [61]. After hemin treatment, HEK293T cells were harvested and their nuclei were isolated to bind with concanavalin A-coated magnetic beads. GST-His_6_-2×HBD was used as an R-loop sensor and incubated with the nuclei. Then anti-His antibody and secondary antibody were added sequentially to tether barcoded protein A-Tn5 transposase. Following Tn5 activation with Mg^2+^, chromatin around GST-His_6_-2×HBD binding sites was cut and integrated with adapters. Tagmented genomic DNA was then extracted and amplified for library preparation and sequencing. **(G-J)** Heatmap and metaplot analysis of R-loop CUT&Tag signals at hemin binding promoters (G and H) and enhancers (I and J) in HEK293T cells with or without hemin treatment. Kolmogorov-Smirnov test (K-S test) was used for the statistical testing and the *p* values were provided.


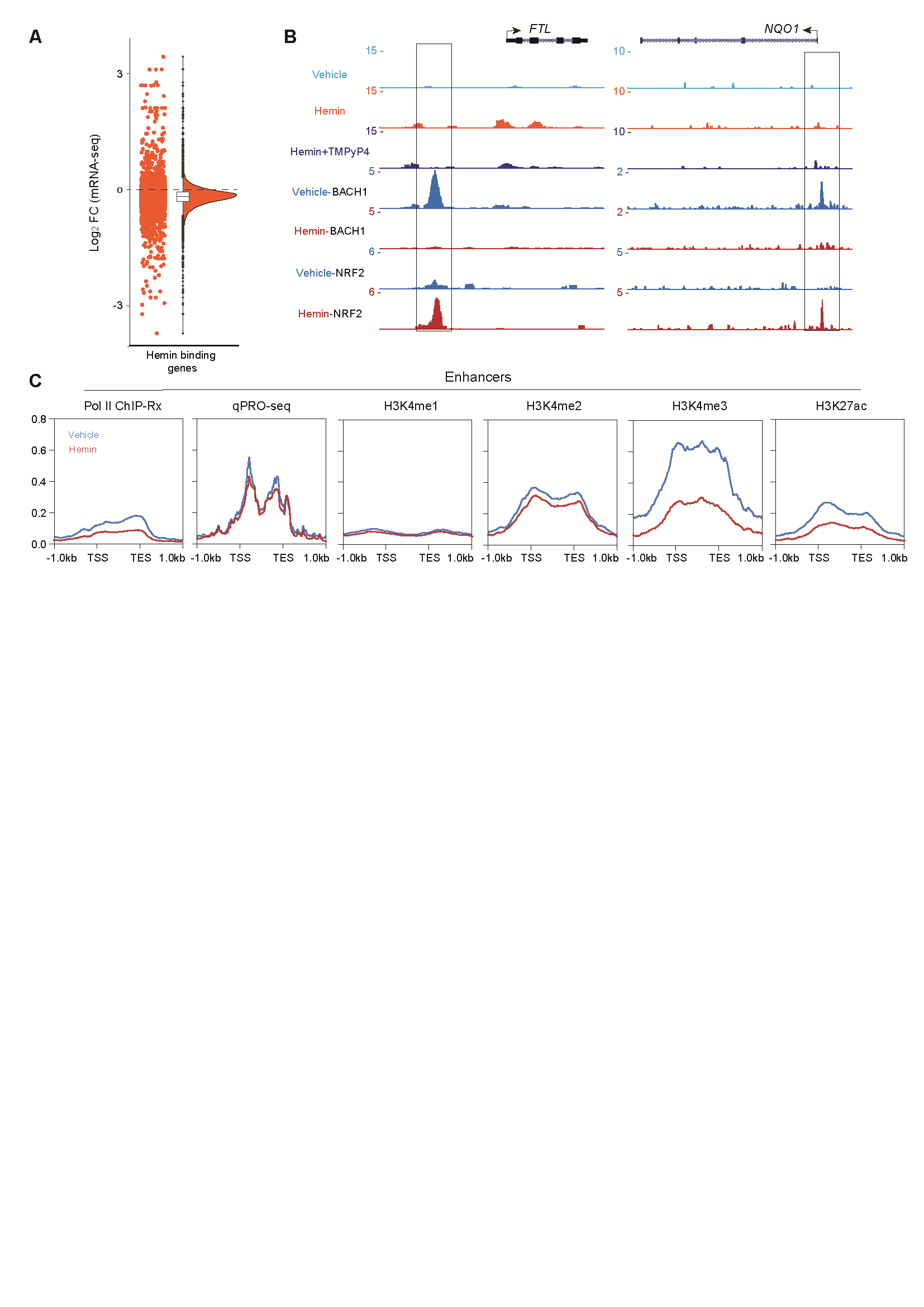


**Figure S4. Decreased enhancer activation at hemin-bound enhancers**

**(A)** Boxplot and violin plots showing the log2 fold changes of hemin binding genes after 6-hour hemin treatment. **(B)** UCSC genome browser snapshots of NRF2, BACH1 ChIP-Rx at well-known NRF2 target loci (*FTL* and *NQO1*). G4 self-biotinylation tracks for vehicle, hemin, and hemin + TMPyP4 are shown for comparison. **(C)** Metaplot analyses of qPRO-seq signals, Pol II, H3K4me1, H3K4me2, H3K4me3, and H3K27ac ChIP-Rx signals at hemin-bound enhancers. Similar to the effects of hemin on promoters, hemin treatment impaired Pol II occupancy, H3K4me3, and H3K27ac levels at hemin-bound enhancers, indicating decreased enhancer activity after hemin treatment.

**Figure S5. Effects of hemin on gene expression in mouse primary hepatocytes**


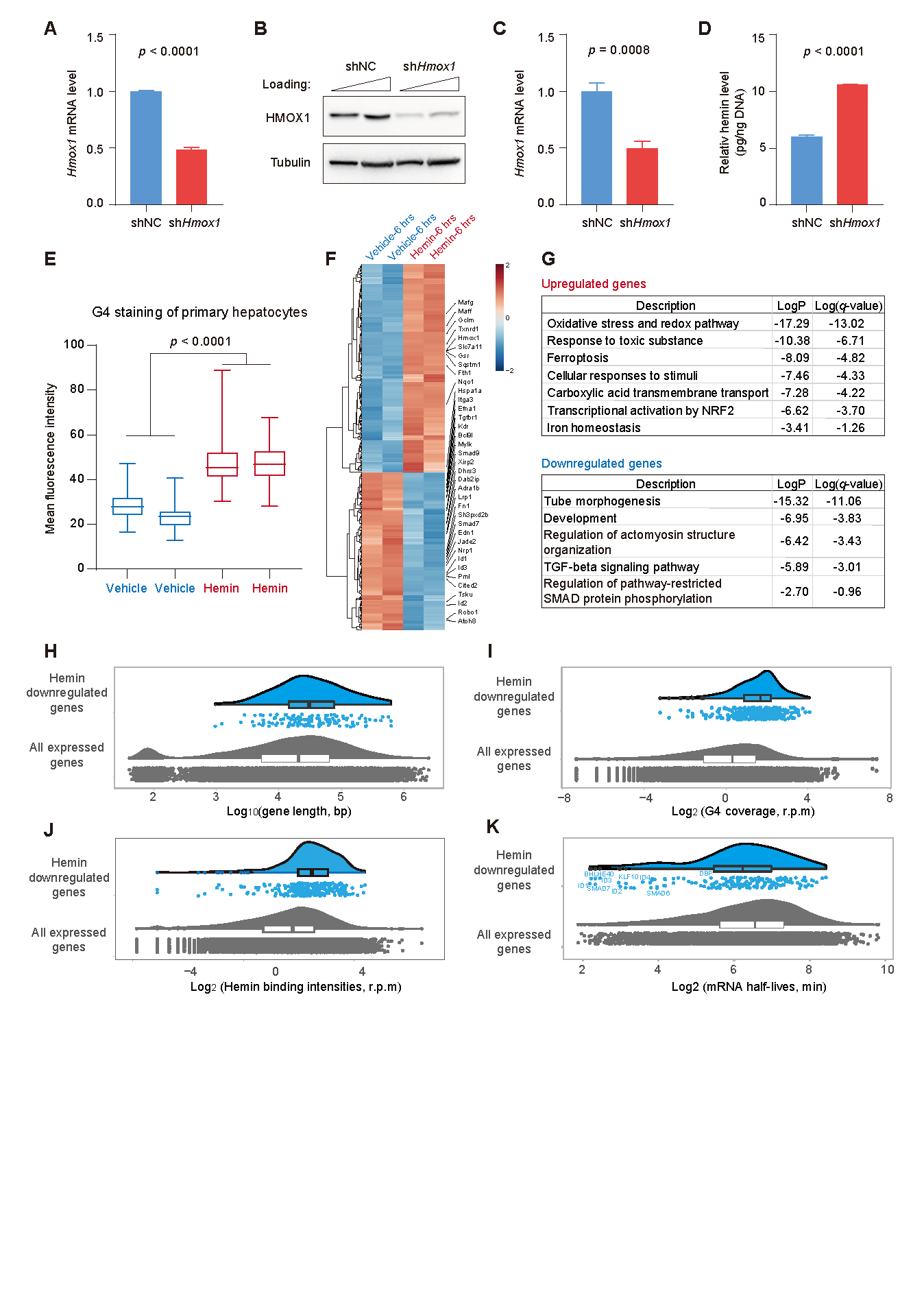


­**(A and B)** Knockdown efficiencies of AAV-sh*Hmox1* in MEF cells with quantitative RT-PCR (A) and western blot (B) analyses. **(C)** Verification of *Hmox1* knockdown in AAV-sh*Hmox1* transduced mouse liver (n=3). **(D)** Quantification of hemin in AAV-sh*Hmox1* transduced mouse liver (n=3). **(E)** Hemin treatment increases G4 signals in primary mouse hepatocytes. For each sample, randomly selected 160 nuclei in 7 fields of vision were used for quantification and normalized by dividing total intensities of BG4-EGFP with DAPI stained nuclear area (https://doi.org/10.6084/m9.figshare.21608127.v1 [88]). **(F)** Heatmap analysis of differentially expressed genes in hemin-treated mouse primary hepatocytes. **(G)** Gene ontology analysis of differentially expressed genes in response to hemin in mouse primary hepatocytes. **(H-K)** Distribution of gene lengths (H), G4 levels after hemin treatment (I), *in vivo* hemin binding intensities (J), and RNA half-lives (K) [71] of hemin-downregulated genes and all expressed genes in HEK293T cells.
