# Supplementary figures and images for: G-quadruplexes sense natural porphyrin metabolites for regulation of gene transcription and chromatin landscapes
Source: Genome Biol. 2022 Dec 15;23:259. doi: 10.1186/s13059-022-02830-8 (PMC9753424; doi:10.1186/s13059-022-02830-8)

Figure S2A:


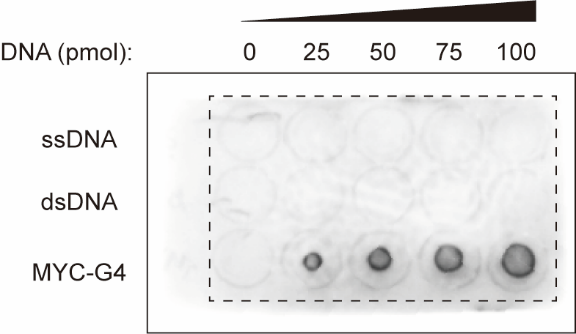


Figure S3A:


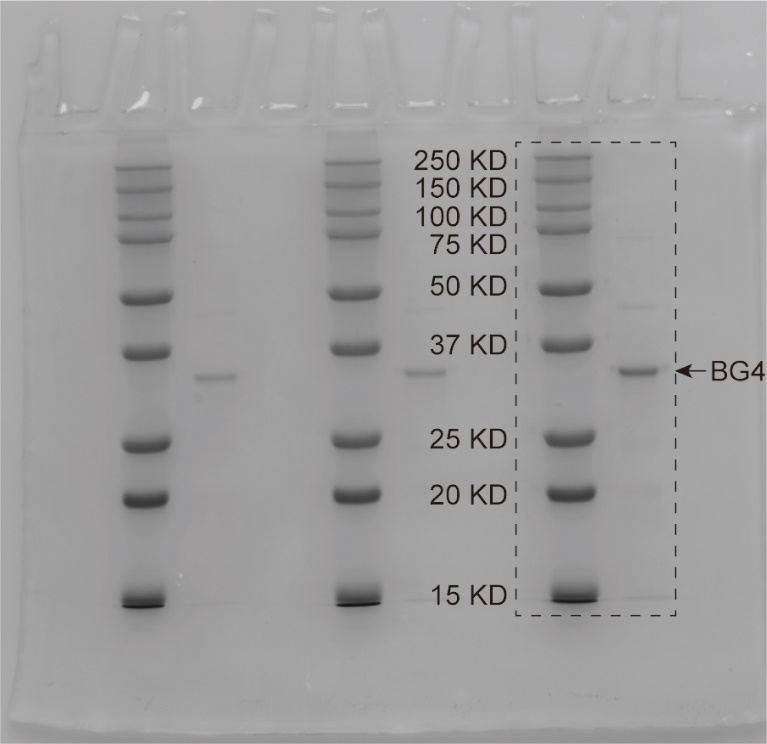


Figure S3B:


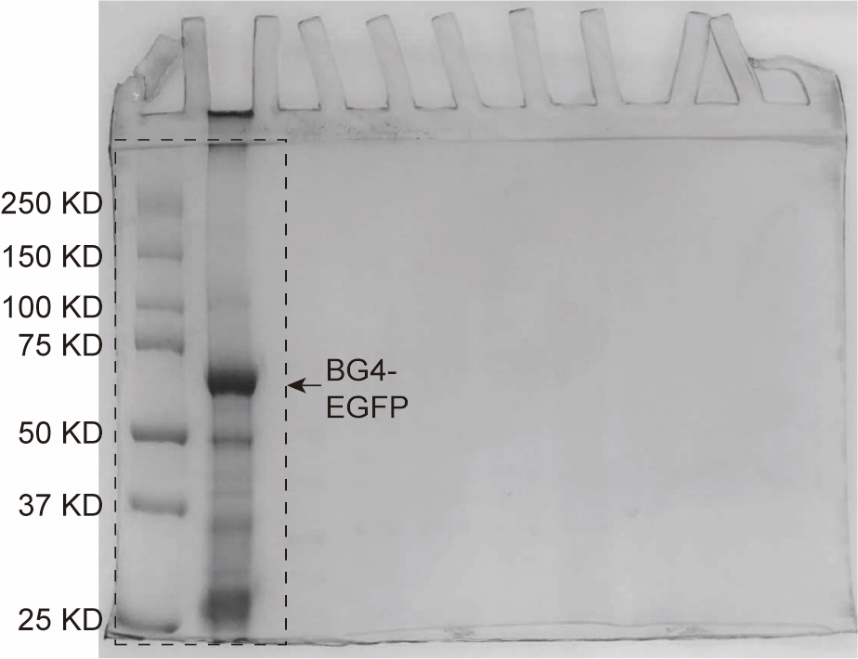


Figure S5B:


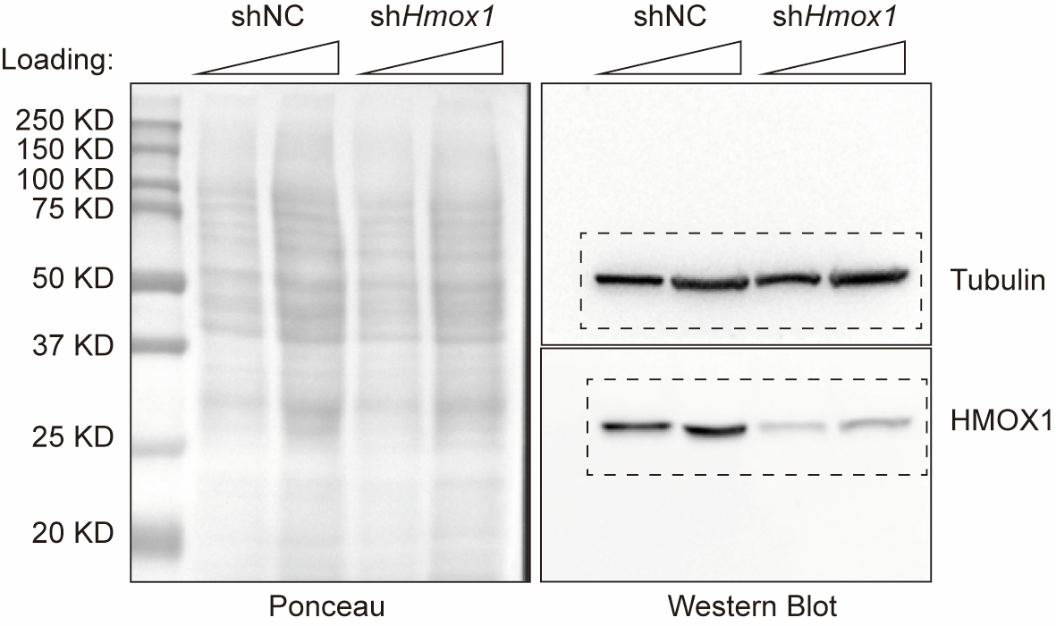

Supplement: Supplementary file 2 — Additional file 2: Uncropped blot images. [file 13059_2022_2830_MOESM2_ESM.docx]
