# Supplementary material for: G-quadruplexes sense natural porphyrin metabolites for regulation of gene transcription and chromatin landscapes
Source: Genome Biol. 2022 Dec 15;23:259. doi: 10.1186/s13059-022-02830-8 (PMC9753424; doi:10.1186/s13059-022-02830-8)
Supplement: Supplementary file 3 — Additional file 3: Review history. [file 13059_2022_2830_MOESM3_ESM.docx]

Review History

**First round of review**

**Reviewer 1**

**Were you able to assess all statistics in the manuscript, including the appropriateness of statistical tests used?** **Y**es, and I have assessed the statistics in my report.

**Comments to author:**

n this manuscript Li et al present the genome-wide interaction of natural porphyrins (hemin) at G-quadruplexes (G4s). They show that part of genome-wide G4s, especially at gene promoters, were also bound by hemin and that treatment with hemin impaired transcription initiation and modulated chromatin markers. On the whole, they propose G4s as sensors of natural porphyrins metabolites in cells and that cell porphyrins in turn regulate transcription and chromatin remodeling through their interaction to G4s.

The manuscript is clearly written, very dense with experiments, the design and results of which look sound.

Comments

1) When characterizing the interaction between G4s and porphyrins in vitro, please add additional G4 sequences with different conformations so to corroborate that porphyrins preferentially bind to parallel G4s. Also, add a control sequence unable to fold in G4. Provide this validation not only for PpIX and hemin, but show also the binding and KD of the synthesised biotin-PEG4-hemin to G4s.

2) As for KD of hemin to MYC G4, please show data when saturation is reached.

3) In Fig 3A-B, what is the contribution of porphyrin fluorescence to the observed signal? Please show images with porphyrin treatment in the absence of EGFP in the same settings.

**Reviewer 2**

**Were you able to assess all statistics in the manuscript, including the appropriateness of statistical tests used?** No, I do not feel adequately qualified to assess the statistics.

**Comments to author:**

The manuscript by Li et al. uses genomic approaches with conventional immortalized cell lines, e.g. 293, to evaluate the impact of hemin on G4 DNA structures and functional relationships between heme mechanisms and G4. While this is an interesting problem, insufficient information was provided to interpret the putative G4 measurements in cells. Statistical considerations related to generating conclusions from overlapping thousands of signals, putatively representing hemin and G4, were not articulated and/or not conducted, raising questions about many of the conclusions. More controls, better descriptions, and additional experiments are required to yield rigorous conclusions regarding potential relationships between hemin and G4 mechanisms. Finally heme-dependent chromatin remodeling genome-wide and heme-dependent transcriptional mechanisms have been described by Liao et al. Cell Reports, June 30, 2020. The data from this study may inform relationships described in the current study.

Specific Comments:

1. Although two prior papers were cited, there was no description of the reagents and methods for quantifying G4 throughout chromatin using CutNTag. Since antibody recognition of these types of DNA structures might not be straightforward (and absolutely specific) in a cellular environment, it is crucial to explain this carefully and provide the requisite controls.

2. The CutNRun and G4Hunter predictions yielded many thousands of putative G4 structures in the cells. The chemically derivatized hemin yielded more than 10000 peaks. Since half localized to promoters, it would not be surprising if these putative hemin binding regions co-localized with a wide spectrum of proteins and motifs, which would not infer a functional relationship between the two. The authors conclude that hemin "can bind a proportion of G4 in the human genome, unveiling a strong correlation between hemin binding sites and G4 at promoter regions". This conclusion is potentially flawed based on the logic articulated above. It would be necessary to eliminate a sequence that forms G4 (without destroying a promoter, enhancer etc) and demonstrate that this eliminates heme binding, rather than to rely upon correlations.

3. Page 7, line 202 - "We treated cells" The specific cells were not stated.

4. Fig. 3A - Resolution is too low to permit rigorous evaluation. It would also be important to include controls to ensure signal specificity.

5. Fig. 3 - hemin and other agents were added to cells and biotin-PEG4 and G4 CutNTag assays were conducted, and the authors described signal changes at promoters of a few genes. However, unless I am missing this, there is no data on the impact of these treatments on transcription of the genes and canonical controls to evaluate promoter functionality.

6. Page 8, line 226 - An R-loop sensor was used to determine if hemin influenced this signal, and the authors stated that it "did not dramatically affect etc". It would be more informative to state the results with statistics, rather than to use "dramatic". Were controls included to ensure that the probe actually detected R-loops?

7. Fig. 4 - Reporters were used, and conclusions were drawn about relationships between hemin, ALA or PPIX and G4 structures, but no strategic mutants were used to assess specificity of any of the effects.

8. Fig. 5 - Where are the transcription (or mRNA) measurements for the genes studied?

9. Page 10, line 298 - What cells were used for the expression analysis?

10. Page 11, 308 - "hemin treatment induced the replacement" First, hemin leads to loss of Bach1 via proteosomal degradation. This is not clear from the description of the experimental results. Secondly, a NRF2 track is shown. It would be important to include positive and negative controls to ensure signal specificity.

11. Page 12 - "blockage of hemin degradation induces liver damage" This biological experiment re-tests what is already know - excess heme is cytotoxic.

**Reviewer 3**

**Were you able to assess all statistics in the manuscript, including the appropriateness of statistical tests used?** No, I do not feel adequately qualified to assess the statistics.

**Comments to author:**

This is an interesting manuscript aimed at investigating the role of Heme and its metabolites in the binding and recognition of G4-structures in chromatin and associated changes in gene-expression and epigenetics. The authors synthesise a biotinilated-Heme, which is then used in Cut&Tag experiments to monitor binding sites of the molecule, revealing some overlap (~40%) with endogenous G4-maps generated with BG4 Cut&Tag and increased G4-sites observed upon treatment with ligand over time. Based on this the authors conclude that Heme is a natural G4-binder capable of modulating gene expression via G4-binding.

There is no doubt that this is an interesting manuscript and that the authors have generated a significant amount of data but this reviewer does not feel that the conclusions presented by the authors are supported at all by the data presented and cannot recommend publication in Genome Biology of the manuscript in its current form. This manuscript is in principle suitable for publication in Genome Biology, but the authors need to significantly revise their conclusions and their data interpretation. Below are my main concerns:

1) The main criticism I have is that the authors use an exogenous Heme ligand (not sure at what concentration this is not specified in the methods) to investigate endogenous heme-binding sites on fixed chromatin. Flushing chromatin with their heme-ligand will simply saturate every possible binding site and will not be reflective of genuine binding sites of the Heme molecule. Essentially this is the same approach used in the past to monitor G4-ligands binding to chromatin or RNA. What the authors can claim is that they have develop a method to study the binding site of a G4-ligand (Heme-based) but they cannot make any conclusions on this interaction being relevant endogenously.

2) On top my main concern above, the overlap with BG4 Cut&Tag is modest, suggesting that most of the binding sites of the ligand are actually not G4s. This might suggest that Heme might have a transcriptional role that is not necessarily only G4-mediated, which is something that should be mentioned in the manuscript.

3) The authors also claim nano molar affinity of their Heme probe agains MYC G4 based on BLI data showed in Figure 1. Besides the fact that they claim a Kd of ~80 nM whilst their lowest concentration tested is 600 nM, which is scientifically flawed; the BLI trace showed clearly shows a very slow Kon and basically the ligand never dissociating (which justifies the very low Kd). This is not how the BLI trace of true binding should look like, a clear example of a true binding curve is in Figure 1K. What showed in Figure 1L is clearly unspecific binding to the surface and cannot be used as a way to assess the affinity of the ligand for the MYC G4.

4) On the same note of point 3 there is no data on the specificity of the ligand for G4s over single stranded and double stranded DNA, which prevents the authors to claim any selectivity of their ligand for G4s and affects the genomics data presented significantly.

5) To further reinforce my unspecific binding concern the genomic traces showed in Figure 3N clearly show similar peaks (with lower intensities) in the Biotin PEG control, which worryingly indicate that what the authors are doing is simply amplifying unspecific binding more that truly pulling down G4s. To verify this the authors should perform a displacement with a canonical G4 ligand in chromatin to show that these sites are truly loss during competition with a G4 binder. This will also allow them to disentangle between Heme-G4 binding and non G4 binding sites.

6) Finally the authors use BG4-EGFP for immuno staining and there is no validation of this BG4 analogue to still be able to bind G4s with selectivity and high affinity. BG4 is a very sensitive antibody and modifications often compromise G4-selectivity. The authors needs to show that this version of the antibody is still suitable for IF experiments.

Minor comments:

7) The authors seems to present as a given the role of G4s in epigenetic regulation and their association as transcriptional repressor, whilst this is something still heavily debated. Their introduction should be more reflective of the real state of the art rather than being biased towards the message they want to send.

**Authors Response**

**Point-by-point responses to the reviewers’ comments:**

***We thank all of the reviewers for their insightful comments and suggestions that have led us to develop a more thorough study that delves deeper into characteristics of hemin biology and its role in transcriptional regulation though G4s as viewed on a genome scale. In the revised manuscript, we demonstrate the preference of hemin for parallel MYC G4-like structures and have developed an assay for identifying endogenous hemin binding sites in cells using a G4 self-biotinylation reaction and CUT&Tag. We have also included additional controls for the in situ biotin-PEG4-hemin capture sequencing and immunofluorescence assays. With these new data in hand, we have reorganized the relevant figures, which we hope the reviewers find are clearer and more comprehensible. Please find our point-by-point response below.***

**Reviewer #1:** In this manuscript Li et al present the genome-wide interaction of natural porphyrins (hemin) at G-quadruplexes (G4s). They show that part of genome-wide G4s, especially at gene promoters, were also bound by hemin and that treatment with hemin impaired transcription initiation and modulated chromatin markers. On the whole, they propose G4s as sensors of natural porphyrins metabolites in cells and that cell porphyrins in turn regulate transcription and chromatin remodeling through their interaction to G4s.

The manuscript is clearly written, very dense with experiments, the design and results of which look sound.

Comments

1) When characterizing the interaction between G4s and porphyrins in vitro, please add additional G4 sequences with different conformations so to corroborate that porphyrins preferentially bind to parallel G4s. Also, add a control sequence unable to fold in G4. Provide this validation not only for PpIX and hemin, but show also the binding and KD of the synthesised biotin-PEG4-hemin to G4s.

***Thanks for these great suggestions. In the revised manuscript, we have added the bio-layer interferometry assays using hemin and a variety of biotin-DNA probes with different conformations. We have tested the binding of hemin with single-stranded DNA, double-stranded DNA, anti-parallel SPB1-G4, mixed parallel/anti-parallel hTELO-G4, and intermolecular hTELO-G4 [1] (Figure S1 and Line 171-175), and found that hemin had no significant binding to these probes in contrast to the strong binding of hemin to parallel MYC-G4 (Fig. 1L). We also performed dot blots of biotin-PEG4-hemin with DNA probes and confirmed that biotin-PEG4-hemin can bind to MYC-G4 in vitro (Fig. S2A and Line 180-181).***

***Figure S1. Bio-layer interferometry analysis of hemin with oligonucleotides***

*(A and B) Bio-layer interferometry analysis of hemin with single-stranded DNA (A) and double-stranded DNA (B). Biotinylated oligonucleotides were immobilized on streptavidin biosensors and incubated with a range of hemin concentrations to measure the response in a Gator instrument. (C-E) Characterization of the interaction between hemin and G4s. Hemin showed no binding with anti-parallel SPB1-G4 (C), mixed parallel/anti-parallel hTELO-G4 (D), or intermolecular hTELO-G4 (E) [1] at the hemin concentration from 0.5 μM to 8 μM.*

2) As for KD of hemin to MYC G4, please show data when saturation is reached.

***We thank the reviewer for raising this point. As also prompted by Reviewer 3, we realized that our previous bio-layer interferometry assay with hemin was flawed since the measured KD was not within the testing-concentration ranges. Therefore, we further optimized the experimental conditions for bio-layer interferometry, which now shows the saturation of hemin (Fig. 1L). With the improved assay, we obtained a KD of 1.52 μM for hemin:MYC-G4 (Line 167-170).***

***Fig. 1L Bio-layer interferometry analysis of MYC-G4 with hemin***

3) In Fig 3A-B, what is the contribution of porphyrin fluorescence to the observed signal? Please show images with porphyrin treatment in the absence of EGFP in the same settings.

***Following the reviewer’s suggestion, we have added the requested negative controls of HeLa cells, which were treated with porphyrins in the absence of BG4- EGFP and captured under the same settings (Fig. S3C and Line 220-222).***

**Reviewer #2:** The manuscript by Li et al. uses genomic approaches with conventional immortalized cell lines, e.g. 293, to evaluate the impact of hemin on G4 DNA structures and functional relationships between heme mechanisms and G4. While this is an interesting problem, insufficient information was provided to interpret the putative G4 measurements in cells. Statistical considerations related to generating conclusions from overlapping thousands of signals, putatively representing hemin and G4, were not articulated and/or not conducted, raising questions about many of the conclusions. More controls, better descriptions, and additional experiments are required to yield rigorous conclusions regarding potential relationships between hemin and G4 mechanisms. Finally heme-dependent chromatin remodeling genome-wide and heme-dependent transcriptional mechanisms have been described by Liao et al. Cell Reports, June 30, 2020. The data from this study may inform relationships described in the current study.

***We sincerely thank the reviewer for these valuable comments and helpful suggestions that have allowed us to improve the manuscript in terms of rigor and scientific impact. In the revised manuscript, we have provided more controls, additional statistical analyses, and have added experiments to better characterize the interaction of hemin with G4 and its roles in transcriptional regulation. To better characterize the hemin binding sites in cells, we used a G4 self-biotinylation reaction with the CUT&Tag method to profile endogenous hemin binding sites in cells. We have also expanded our introduction section that we hope provides a better overview of the G4 and hemin research field, which should better clarify the contributions of our study. Please find our point-by-point response below.***

Specific Comments:

1. Although two prior papers were cited, there was no description of the reagents and methods for quantifying G4 throughout chromatin using CutNTag. Since antibody recognition of these types of DNA structures might not be straightforward (and absolutely specific) in a cellular environment, it is crucial to explain this carefully and provide the requisite controls.

***In the revised manuscript, we have added the reagents and method information in the Methods (Line 675-687). Following the reviewer’s suggestions, we also added the IgG negative controls in the G4-CUT&Tag and R-loop CUT&Tag analysis (Fig. 3J-M and Fig. S3G-J).***

2. The CutNRun and G4Hunter predictions yielded many thousands of putative G4 structures in the cells. The chemically derivatized hemin yielded more than 10000 peaks. Since half localized to promoters, it would not be surprising if these putative hemin binding regions co-localized with a wide spectrum of proteins and motifs, which would not infer a functional relationship between the two. The authors conclude that hemin "can bind a proportion of G4 in the human genome, unveiling a strong correlation between hemin binding sites and G4 at promoter regions". This conclusion is potentially flawed based on the logic articulated above. It would be necessary to eliminate a sequence that forms G4 (without destroying a promoter, enhancer etc) and demonstrate that this eliminates heme binding, rather than to rely upon correlations.

***We are grateful for the reviewer’s insightful comments. We have extensively revised the Fig. 2 to show the in situ biotin-PEG4-hemin binding and added the data of mapping endogenous hemin binding sites in cells. First, to test the specificity of biotin-PEG4-hemin to G4 in the chromatin context, we used a canonical G4 ligand TMPyP4 to displace Biotin-PEG-hemin in the biotin-PEG4-hemin capture sequencing assays, according to the suggestions by Reviewer 3. We found that the G4 ligand TMPyP4 effectively reduced signals at Biotin-PEG-hemin binding sites (Fig 2D-E and Line 187-192). Second, we have taken advantage of the intrinsic peroxidase activity of G4-hemin complexes. Treating G4-hemin complexes with hydrogen peroxide and biotin tyramide could lead to efficient covalent biotinylation of G4 in vitro [2], which has been shown to be highly specific and spatially-restricted [3]. Based on this reaction, we mapped the self-biotinylation of G4-hemin and profiled the endogenous hemin binding sites at G4 (Fig. 2F-G and Line 199-206). The G4 ligandTMPyP4 was used to further validate these*** ***endogenous hemin binding sites in HEK293T cells (Line 206-214).***

***Fig. 2D-E Analysis of biotin-PEG4-hemin binding signals and G4 CUT&Tag signals***

*(D) UCSC genome browser snapshots of in situ biotin-PEG4-hemin binding signals and G4 CUT&Tag signals in HEK293T cells. TMPyP4 was used as a competitor for biotin-PEG4-hemin. (E) Heatmap and metaplot analysis of biotin-PEG4-hemin binding signals and G4 CUT&Tag signals.*

***Fig. 2F-G Profiling of hemin binding sites in cells using G4 self-biotinylation reaction and CUT&Tag.***

*(F) UCSC genome browser snapshots of G4 self-biotinylation signals in HEK293T cells in the presence or absence of the TMPyP4 competitor. (G) Heatmaps of G4 self-biotinylation in the HEK293T after treatment of vehicle or hemin. With TMPyP4 competitor, we characterized 8,195 G4 self-biotinylation peaks which were sensitive to TMPyP4 treatment.*

3. Page 7, line 202 - "We treated cells" The specific cells were not stated.

***Fixed. HeLa cells were used here.***

4. Fig. 3A - Resolution is too low to permit rigorous evaluation. It would also be important to include controls to ensure signal specificity.

***Fixed, we have added the negative controls, which were treated with porphyrins in the absence of BG4- EGFP in the same settings (Fig. S3C and Line 220-222).***

5. Fig. 3 - hemin and other agents were added to cells and biotin-PEG4 and G4 CutNTag assays were conducted, and the authors described signal changes at promoters of a few genes. However, unless I am missing this, there is no data on the impact of these treatments on transcription of the genes and canonical controls to evaluate promoter functionality.

***Thanks for the reviewer’s comments. We have improved our writing in this section (Line 305-309) and included additional experiments. In the revised manuscript, we used the in vivo hemin binding peaks in the absence or presence of TMPyP4 and characterized 5,858 promoters and 612 enhancers. To test the effects of hemin on promoter functionality, we performed Pol II ChIP-Rx, TFIIB ChIP-Rx, and qPRO-seq and showed that hemin impairs transcription initiation of these promoters (Fig. 4). Moreover, to measure the transcriptional output and minimize potential secondary effects, we treated HEK293T cells with hemin for 6 hours (Fig. 6) and performed mRNA-seq. Although these 5,858 genes showed a reduction trend (Fig. S4A) within this short period of hemin treatment, we only identified a few hundred significantly downregulated genes, which are generally associated with higher G4 levels, higher hemin binding, and shorter mRNA half-lives (Fig. S5H-K).***

6. Page 8, line 226 - An R-loop sensor was used to determine if hemin influenced this signal, and the authors stated that it "did not dramatically affect etc". It would be more informative to state the results with statistics, rather than to use "dramatic". Were controls included to ensure that the probe actually detected R-loops?

***In the revised manuscript, we have provided the IgG control in the R-loop CUT&Tag analysis (Fig. S3G and S3I). We apologize that we mislabeled the metaplot in the previous version of the manuscript and we have updated the metaplots with the hemin-binding sites from in vivo G4-hemin self-biotinylation assays (Fig. 2). We found that hemin treatment did not increase the R-loop signals at hemin binding promoters and enhancers (Fig. S3H and S3J). Instead, hemin slightly but significantly decreased the R-loop CUT&Tag signals at hemin-binding promoters and enhancers (Fig. S3H and S3J), which may be caused by the decrease Pol II occupancy after hemin treatment (Fig. 4). We have revised the relevant statements in the manuscript (Line 242-245).***

7. Fig. 4 - Reporters were used, and conclusions were drawn about relationships between hemin, ALA or PPIX and G4 structures, but no strategic mutants were used to assess specificity of any of the effects.

***Thanks for the reviewer’s suggestions. We established the luciferase-reporting system to test the effects of porphyrins on different promoter sequences. Specifically, we cloned the plasmids with insertion of large fragments of promoter DNA, which are G-rich and with lengths between 400-600 bp. However, given that there are numerous G sites which potentially participate in G4 formation and the G4 conformations are highly variable, it is very difficult to obtain strategic mutants by mutating one or a few bases in this system.***

8. Fig. 5 - Where are the transcription (or mRNA) measurements for the genes studied?

***Pol II occupancy, TFIIB occupancy, and qPRO-seq signals at these genes all indicate transcriptional impairment (Fig. 4).***

9. Page 10, line 298 - What cells were used for the expression analysis?

***Fixed. HEK293T cells were used for the mRNA-seq analysis.***

10. Page 11, 308 - "hemin treatment induced the replacement" First, hemin leads to loss of Bach1 via proteosomal degradation. This is not clear from the description of the experimental results. Secondly, a NRF2 track is shown. It would be important to include positive and negative controls to ensure signal specificity.

***We agree with the reviewer and we have modified our statements (Line 325-327). In addition, we now provided tracks of NRF2 binding at the known NQO1 and FTL loci to show the specificity of NRF2 ChIP-seq (Fig. S4B).***

11. Page 12 - "blockage of hemin degradation induces liver damage" This biological experiment re-tests what is already know - excess heme is cytotoxic.

***Thanks for the reviewer’s suggestion. In the revised manuscript, we have revised this section and shift focus to the altered gene expression profiles of mouse primary hepatocytes by hemin.***

**Reviewer #3:** This is an interesting manuscript aimed at investigating the role of Heme and its metabolites in the binding and recognition of G4-structures in chromatin and associated changes in gene-expression and epigenetics. The authors synthesise a biotinilated-Heme, which is then used in Cut&Tag experiments to monitor binding sites of the molecule, revealing some overlap (~40%) with endogenous G4-maps generated with BG4 Cut&Tag and increased G4-sites observed upon treatment with ligand over time. Based on this the authors conclude that Heme is a natural G4-binder capable of modulating gene expression via G4-binding.There is no doubt that this is an interesting manuscript and that the authors have generated a significant amount of data but this reviewer does not feel that the conclusions presented by the authors are supported at all by the data presented and cannot recommend publication in Genome Biology of the manuscript in its current form. This manuscript is in principle suitable for publication in Genome Biology, but the authors need to significantly revise their conclusions and their data interpretation. Below are my main concerns:

***We thank the reviewer for the insightful comments and helpful suggestions that have stimulated us to carry out additional experiments to evaluate heme binding in cells, improve existing assays and provide better context in our discussion of G4 biology.***

1) The main criticism I have is that the authors use an exogenous Heme ligand (not sure at what concentration this is not specified in the methods) to investigate endogenous heme-binding sites on fixed chromatin. Flushing chromatin with their heme-ligand will simply saturate every possible binding site and will not be reflective of genuine binding sites of the Heme molecule. Essentially this is the same approach used in the past to monitor G4-ligands binding to chromatin or RNA. What the authors can claim is that they have develop a method to study the binding site of a G4-ligand (Heme-based) but they cannot make any conclusions on this interaction being relevant endogenously.

***We sincerely thank the reviewer for pointing this out and we completely agree with the reviewer’s comments. To map the endogenous hemin binding sites, we took advantage of the intrinsic peroxidase activity of G4-hemin complexes. Since treating G4-hemin complexes in the presence of hydrogen peroxide and biotin tyramide can lead to efficient covalent biotinylation of G4 in vitro is a highly specific and spatially-restricted manner [2, 3], we developed an assay combining G4 self-biotinylation and CUT&Tag approach to map the hemin binding sites in cells (Fig. 2F, Response to Reviewer 2 point 2). This assay generated better signals than biotin-PEG4-hemin capture sequencing method. Furthermore, we used the G4 ligand TMPyP4 to compete with hemin binding and identified the genome-wide hemin binding sites for use in downstream analyses.***

2) On top my main concern above, the overlap with BG4 Cut&Tag is modest, suggesting that most of the binding sites of the ligand are actually not G4s. This might suggest that Heme might have a transcriptional role that is not necessarily only G4-mediated, which is something that should be mentioned in the manuscript.

***Thanks for these comments. In the revised manuscript, we have addressed this issue in the discussion section (Line 418-424).***

3) The authors also claim nano molar affinity of their Heme probe agains MYC G4 based on BLI data showed in Figure 1. Besides the fact that they claim a Kd of ~80 nM whilst their lowest concentration tested is 600 nM, which is scientifically flawed; the BLI trace showed clearly shows a very slow Kon and basically the ligand never dissociating (which justifies the very low Kd). This is not how the BLI trace of true binding should look like, a clear example of a true binding curve is in Figure 1K. What showed in Figure 1L is clearly unspecific binding to the surface and cannot be used as a way to assess the affinity of the ligand for the MYC G4.

***We are grateful to the reviewer for pointing this out. We agree that our previous BLI assay using hemin and MYC-G4 was flawed due to unspecific binding. In the revised manuscript, we optimized the BLI assays with hemin and showed that hemin was bound to MYC-G4 with a KD of 1.52 μM (Fig. 1L, Response to Reviewer 1 point 2).***

4) On the same note of point 3 there is no data on the specificity of the ligand for G4s over single stranded and double stranded DNA, which prevents the authors to claim any selectivity of their ligand for G4s and affects the genomics data presented significantly.

***In the revised manuscript, we have added the bio-layer interferometry assays using hemin and a variety of DNA probes with different conformations. We have tested the binding of hemin with single-stranded DNA, double-stranded DNA, anti-parallel SPB1-G4, mixed parallel/anti-parallel hTELO-G4, and intermolecular hTELO-G4 [1] (Figure S1, Line 171-175), which showed that hemin had no binding with these probes within the tested hemin concentration range of 0.5 μM to 8 μM.***

5) To further reinforce my unspecific binding concern the genomic traces showed in Figure 3N clearly show similar peaks (with lower intensities) in the Biotin PEG control, which worryingly indicate that what the authors are doing is simply amplifying unspecific binding more that truly pulling down G4s. To verify this the authors should perform a displacement with a canonical G4 ligand in chromatin to show that these sites are truly loss during competition with a G4 binder. This will also allow them to disentangle between Heme-G4 binding and non G4 binding sites.

***Thanks for the great suggestions. In the revised manuscript, we have used the G4 ligand TMPyP4 as a competitor of hemin in both the biotin-PEG4-hemin capture sequencing and G4 self-biotinylation assays. Following these suggestions, we further used the TMPyP4 to characterize the endogenous hemin binding sites in the genome (Fig 2 and Line 187-192).***

6) Finally the authors use BG4-EGFP for immunostaining and there is no validation of this BG4 analogue to still be able to bind G4s with selectivity and high affinity. BG4 is a very sensitive antibody and modifications often compromise G4-selectivity. The authors needs to show that this version of the antibody is still suitable for IF experiments.

***Thanks for the comments. The specificity of BG4-EGFP has been confirmed in our previous study [4]*** ***, which we now describe in the revised manuscript (Line 517-518).***

***Specificity of BG4 and BG4-EGFP to G4s [4]***

Minor comments:

7) The authors seems to present as a given the role of G4s in epigenetic regulation and their association as transcriptional repressor, whilst this is something still heavily debated. Their introduction should be more reflective of the real state of the art rather than being biased towards the message they want to send.

***Thanks for the comments. We agree with the reviewer’s criticism and we have modified our introduction section accordingly (Line 81-88, line 99-102).***

References:

1.	Biffi G, Tannahill D, McCafferty J, Balasubramanian S: **Quantitative visualization of DNA G-quadruplex structures in human cells.** *Nat Chem* 2013, **5:**182-186.

2.	Einarson OJ, Sen D: **Self-biotinylation of DNA G-quadruplexes via intrinsic peroxidase activity.** *Nucleic Acids Res* 2017, **45:**9813-9822.

3.	Lat PK, Liu K, Kumar DN, Wong KKL, Verheyen EM, Sen D: **High specificity and tight spatial restriction of self-biotinylation by DNA and RNA G-Quadruplexes complexed in vitro and in vivo with Heme.** *Nucleic Acids Res* 2020, **48:**5254-5267.

4.	Li C, Wang H, Yin Z, Fang P, Xiao R, Xiang Y, Wang W, Li Q, Huang B, Huang J, Liang K: **Ligand-induced native G-quadruplex stabilization impairs transcription initiation.** *Genome Res* 2021, **31:**1546-1560.

**Second round of review**

**Reviewer 1**

The authors have satisfactorily answered my previous comments.

**Reviewer 2**

The revisions have appropriately addressed most of my comments.

Additional points include:

page 2, line 38 - It should be made clear that this analysis tests where heme binds crosslinked chromatin - not, where heme binds in cells.

same comment for the subtitle of page 7, line 177 and page 15, line 418.

page 8, line 215 "bind a proportion" is not particularly informative - please be more specific.

page 9, line 243 - "heme has a modest but significant effect on" This statement does not describe what heme does in this context.

page 11, line 303 - From a biological perspective, it will not be clear why the 293T cells were used. This should be articulated.

page 11, line 306 - "hemin-bound genes showed tended" This statement is ambigious and should be revised with more specificity.

page 12, line 322 - "canonical pathway" requires a reference

page 13, line 351 - Is "characterization of" required?

The authors argued for the merits of the luciferase reporter assay and difficulties in inactivating endogenous elements and did not address this important point with experimental analysis. Based on this, it would be important to highlight the limitations of the reporter gene assay i.e. lack of utility for recapitualing normal transcriptional mechanisms in chromatin, many copies of template per cell etc.

**Reviewer 3**

The authors have greatly improved their manuscript since their first submission, especially by including Biotinilated cut&tag.

There are still a couple of point that need addressing.

1) The main one is on the porphyrin-displacement measurement with TMPyP4. This is a porphyrin based ligand and would be biased for this assay and to support the authors conclusion. A non porphyrin ligand like PDS or PhenDC3 should be used to really support the authors conclusion.

2) The Kd values should be reported with standard deviation, also what are the Kd values for ssDNA and dsDNA?

Subject to the above I am supportive of publication in genome biology

**Authors Response**

**Point-by-point responses to the reviewers’ comments:**

***We thank all three reviewers for their comments and suggestions which have greatly improved our manuscript. In this revised manuscript, we have included the PDS group as an additional control in the G4 self-biotinylation reaction and CUT&Tag, and added the standard deviations of KD values for PpIX and Hemin. Besides, according to the Reviewers’ suggestion, we have clarified our statements and expanded our discussion regarding the reporter gene assays. Please find our point-by-point response below.***

Reviewer #1: The authors have satisfactorily answered my previous comments.

***We sincerely thank the reviewer for the suggestions and comments.***

Reviewer #2: The revisions have appropriately addressed most of my comments.
Additional points include:

page 2, line 38 - It should be made clear that this analysis tests where heme binds crosslinked chromatin - not, where heme binds in cells. same comment for the subtitle of page 7, line 177 and page 15, line 418.
***Thanks for the suggestions, we have modified the sentences and stated the chromatin context. Please refer to page 2, line 39, page 7, line 177 and page 15, line 424.***

page 8, line 215 "bind a proportion" is not particularly informative - please be more specific.
***Fixed, we have specified the overlapping between hemin binding and G4 peaks (Line 215-216).***

page 9, line 243 - "heme has a modest but significant effect on" This statement does not describe what heme does in this context.

***Fixed, we have clarified the statement here (Line 243).***

page 11, line 303 - From a biological perspective, it will not be clear why the 293T cells were used. This should be articulated.

***Following the reviewer’s suggestion, we have added the explanation for why 293T was used for the RNA-seq analysis (Line 301-305).***

page 11, line 306 - "hemin-bound genes showed tended" This statement is ambigious and should be revised with more specificity.

***Fixed (Line 306).***

page 12, line 322 - "canonical pathway" requires a reference
***Fixed.***

page 13, line 351 - Is "characterization of" required?
***Thanks for the suggestion. We have deleted these redundant words.***

The authors argued for the merits of the luciferase reporter assay and difficulties in inactivating endogenous elements and did not address this important point with experimental analysis. Based on this, it would be important to highlight the limitations of the reporter gene assay i.e. lack of utility for recapitulating normal transcriptional mechanisms in chromatin, many copies of template per cell etc.

***Following the reviewer’s comments, we have added the discussion of reporter gene assays in the discussion section and described the potential limits of this assay (Line 409-413)***

Reviewer #3: The authors have greatly improved their manuscript since their first submission, especially by including Biotinilated cut&tag.

There are still a couple of point that need addressing.

1) The main one is on the porphyrin-displacement measurement with TMPyP4. This is a porphyrin based ligand and would be biased for this assay and to support the author’s conclusion. A non-porphyrin ligand like PDS or PhenDC3 should be used to really support the author’s conclusion.

***Thanks for these great suggestions. In the revised manuscript, we have included the PDS group in the G4 self-biotinylation assay to further support our statement. PDS could also displace hemin genome-wide as shown in Fig. 2F-2G. Besides, we also included additional track examples in Fig. 3J-3K and Fig. 3N and added the related information in the figure legends and method section. The raw data and processed bigwig file have been uploaded to the GEO repository with accession number GSE198658 and the token qpwfaqegzzotxav.***

2) The Kd values should be reported with standard deviation, also what are the Kd values for ssDNA and dsDNA？

***The standard deviations of KD values have been added for PpIX and hemin in the Fig. 1K-1L. Besides, we also added the errors for the Kon and Koff values. Since ssDNA and dsDNA did not show binding with hemin within the concentration between 8 μM and 0.5 μM (Fig. S1A-S1B), we could not obtain reliable KD values for ssDNA and dsDNA.***

Subject to the above I am supportive of publication in genome biology

**Third round of review**

**Reviewer 3**

The authors have now fully addressed my concerns and I am supportive for publication in Genome Biology
